# Maternal intake of folate and folic acid during pregnancy and markers of male fecundity: A population‐based cohort study

**DOI:** 10.1111/andr.13364

**Published:** 2022-12-26

**Authors:** Anne Gaml‐Sørensen, Nis Brix, Birgit Bjerre Høyer, Sandra Søgaard Tøttenborg, Karin Sørig Hougaard, Jens Peter Ellekilde Bonde, Pernille Jul Clemmensen, Andreas Ernst, Linn Håkonsen Arendt, Sjurdur Frodi Olsen, Charlotta Granström, Tine Brink Henriksen, Gunnar Toft, Cecilia Høst Ramlau‐Hansen

**Affiliations:** ^1^ Department of Public Health Research Unit for Epidemiology Aarhus University Aarhus C Denmark; ^2^ Department of Clinical Genetics Aarhus University Hospital Aarhus N Denmark; ^3^ Department of Occupational and Environmental Medicine Bispebjerg and Frederiksberg Hospital University of Copenhagen Copenhagen NV Denmark; ^4^ Open Patient data Explorative Network Odense University Hospital Odense Denmark; ^5^ Department of Public Health University of Copenhagen Copenhagen K Denmark; ^6^ National Research Centre for the Working Environment Copenhagen Oest Denmark; ^7^ Department of Urology Aarhus University Hospital Aarhus N Denmark; ^8^ Department of Obstetrics and Gynecology Aarhus University Hospital Aarhus N Denmark; ^9^ Department of Epidemiology Research Statens Serum Institut Copenhagen S Denmark; ^10^ Department of Clinical Medicine Aarhus University Aarhus C Denmark; ^11^ Department of Paediatrics Aarhus University Hospital Aarhus N Denmark; ^12^ Steno Diabetes Center Aarhus Aarhus University Hospital Aarhus N Denmark

**Keywords:** dietary supplement, male infertility, prenatal exposure, reproductive hormones, semen quality, testes volume

## Abstract

**Background:**

Poor male fecundity is of concern, and a prenatal origin has been proposed. Folate, a methyl donor involved in DNA methylation, is essential for normal fetal development by regulating gene expression during different periods of fetal development. Thus, prenatal exposure to low maternal folate intake might have a programing function of the developing reproductive organs.

**Objectives:**

To examine the association between maternal intake of folate from diet and folic acid from supplements during pregnancy and markers of fecundity in young men.

**Materials and methods:**

We conducted a follow‐up study using a Danish mother–son cohort of 787 young men born 1998–2000. Percentage differences in semen characteristics, testes volume, and reproductive hormone levels were analyzed according to total folate calculated as dietary folate equivalents from diet and supplements in midpregnancy, using multivariable negative binomial regression models. Total folate was analyzed in quintiles, continuous per standard deviation decrease (SD: 318 μg/day) and as restricted cubic splines.

**Results:**

Low maternal intake of total folate was associated with lower total sperm count (−5% (95% confidence intervals [CI]: −11%; 2%)), a lower proportion of non‐progressive and immotile spermatozoa (−5% [95% CI: −8%; −3%]), and lower testes volume (−4% [95% CI: −6%; −2%]) per SD decrease in total folate intake. Spline plots supported these findings.

**Discussion:**

The finding of a lower proportion of non‐progressive and immotile spermatozoa, and hence a higher proportion of motile spermatozoa, in men of mothers with a lower intake of total folate in midpregnancy was surprising and may be a chance finding.

**Conclusion:**

Lower maternal intake of total folate in midpregnancy was associated with lower sperm count and lower testes volume, however, also with a lower proportion of non‐progressive and immotile spermatozoa in adult men. Whether this actually affects the ability to obtain a pregnancy warrants further investigation.

## 1 INTRODUCTION

Poor male fecundity is of concern in most developed countries.^1^, ^2^ About 40% of Danish men have suboptimal sperm counts, and the tendency is similar in other Western countries.^1^, ^2^, ^3^, ^4^ Low sperm count is associated with low fecundity, longer time to pregnancy, and an increase in the need of medically assisted reproduction to achieve pregnancy.^5^, ^6^ A prenatal origin of poor male fecundity has been suggested.^7^, ^8^ The exact mechanism remains to be fully elucidated, but studies indicate an importance of fetal programming for later male fecundity.^2^, ^9^

The hypothalamic–pituitary–gonadal axis and the reproductive organs may be programmed through DNA methylations, which are epigenetic modifications critical for regulation of gene expression during fetal development.^9^, ^10^ Folate, a water‐soluble vitamin (B_9_) that includes both folate in diet (mostly in green vegetables, liver, fruit, peas, and beans) and synthetic folic acid from supplements and fortified foods,^11^ plays a crucial role in regulating DNA methylation during fetal development.^12^, ^13^ Low prenatal exposure to folic acid has been associated with altered DNA methylation patterns that persist into adulthood.^14^ Following a series of biochemical events in the folate‐dependent one‐carbon metabolism, folate or folic acid ultimately results in the synthesis of the universal methyl donor *S*‐adenosylmethionine that supplies the methyl groups needed for DNA methylations. The folate‐dependent one‐carbon metabolism is dependent on the enzyme methylenetetrahydrofolate reductase (MTHFR).^10^, ^13^, ^15^ Interestingly, polymorphisms in the gene encoding the enzyme MTHFR have been associated with male infertility, possibly through DNA hypomethylations.^16^, ^17^, ^18^ Moreover, methylation dysregulation of testicular DNA and during the genesis of male germ cells may be associated with later male infertility.^16^, ^17^, ^18^

Therefore, the potential association between prenatal exposure to folate and markers of male fecundity warrants further investigation. To date, only one cohort study of 347 Danish men has been conducted in this field and found no association between maternal intake of folic acid supplements and semen quality.^19^ We aimed to investigate the association between maternal intake of total folate from diet and folic acid from supplements during pregnancy and markers of male fecundity in a large sample of young men.

## 2 MATERIALS AND METHODS

**FIGURE 1:**
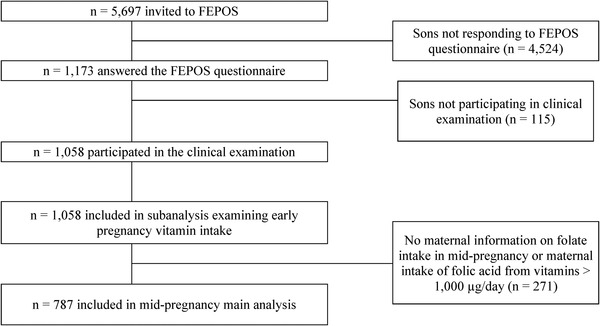
Flowchart. Flowchart of the inclusion of study participants, the Fetal Programming of Semen quality (FEPOS) cohort, nested within the Danish National Birth Cohort (DNBC) 1998–2019, Denmark

A sample of sons of mothers enrolled between 1998 and 2000 in the Danish National Birth Cohort (DNBC)^20^ was invited to participate in the Fetal Programming of Semen quality (FEPOS) cohort between March 2017 and December 2019.^21^ In the DNBC enrolment form, the mother provided information on intake of supplements, when she was around 8 weeks pregnant. In the first DNBC interview, the mother completed a comprehensive baseline questionnaire on health and health behavior, when she was around 16 weeks pregnant. In order for their sons to be eligible for enrolment in the FEPOS cohort, the mothers should have provided a blood sample during pregnancy and participated in the two pregnancy interviews in the DNBC. The recruitment is described in detail elsewhere.^21^ In total, 5697 adult sons were invited of which 1058 participated (19%) in a clinical visit, delivered a semen and a blood sample, and performed self‐measurement of their testes volume. Of these, 787 (74%) had mothers, who also had completed a food frequency questionnaire (FFQ), when she was around 25 weeks pregnant. Accordingly, the final study population consisted of 787 mother–son pairs with information on both maternal intake of folate and folic acid from the FFQ and markers of male fecundity (Figure 1).

### 2.1 Exposure

The main exposure was maternal intake of total folate measured as dietary folate equivalents (DFEs) in μg/day, which was obtained from the FFQ answered in midpregnancy. Mothers provided information on frequency of intake of 360 items of food and beverages and intake of supplements.^22^ Based on estimated standard recipes and standard portion sizes, folate intake from diet was extracted using the Danish food composition tables^23^ described in detail elsewhere.^22^ Folic acid intake from supplements was extracted based on the name and producer of the reported supplement, if any, in addition to estimated daily dose taken of the supplements during the 4 weeks prior to completion of the questionnaire. To avoid confounding by indication, we excluded mothers with an intake of folic acid from supplements above 1000 μg/day (<5 mothers) from the analyses, as 1000 μg/day may be considered the upper limit for intake unless prescribed by a medical doctor.^24^ Total intake of folate and folic acid for the 4‐week period was derived from those two sources and expressed as DFEs to account for the increased bioavailability of folic acid (DFE = folate from diet + folic acid from supplements × 1.7).^11^ The FFQ can be found at https://www.dnbc.dk/data‐available/food‐frequency‐questionnaire.

Information on early pregnancy intake of supplements was obtained for all pregnant women in the DNBC enrolment form and used in a subanalysis and in calculating inverse probability of selection weights.

### 2.2 Markers of male fecundity

Clinical visits took place at Department of Occupational and Environmental Medicine at Bispebjerg Hospital in Copenhagen and Department of Occupational Medicine at Aarhus University Hospital in Aarhus and were conducted by one of two trained medical laboratory technicians. The participants were asked to abstain from sexual activities for at least 2 days before collecting the sample. Semen samples were collected at the residence (18%) or at the clinics (82%). Participants noted the actual abstinence time and provided information about time of sampling and potential spillage during semen sample collection. Semen volume was measured by the weighing of the sample in the pre‐weighed container. The proportion of progressive, non‐progressive and immotile spermatozoa and sperm concentration was determined manually. Total sperm count was calculated by multiplication of sperm concentration and semen volume. Sperm morphology was classified normal or abnormal according to the strict criteria.^25^ Percentage of spermatozoa with DNA fragmentation (DNA fragmentation index [DFI]) and high‐stainability DNA (HDS) was measured on thawed semen samples using sperm chromatin structure assay (SCSA), as described in detail in Evenson.^26^ This was analyzed at the Centre of Reproductive Medicine in Malmö, Sweden. All semen quality analyses were performed according to the World Health Organization's recommendations from 2010,^27^ and methods were continuously quality controlled and met given standards for semen quality measures.^21^

Self‐measurements of testes volume were performed by use of a Prader orchidometer, a method validated by Ramlau‐Hansen et al.^28^ We calculated and estimated associations with the average volume of the two testes.

All analyses of reproductive hormones were conducted at the Department of Clinical Biochemistry, Aarhus University Hospital, Denmark in non‐fasting venous blood samples collected at the clinical examination. Testosterone (limit of detection (LOD): 0.12 nmol/L) and estradiol (LOD: 15 pmol/L) were analyzed using liquid chromatography‐tandem mass spectrometry. Sex‐hormone binding globulin (LOD: 0.350 nmol/L), follicle‐stimulating hormone (FSH) (LOD: 0.1 IU/L), and luteinizing hormone (LH) (LOD: 0.1 IU/L) were measured using immunoassays (Cobas 8000 e602; Roche Diagnostics, Mannheim, Germany). We imputed values below the LODs by LOD/√2 (0.1% for FSH and LH and 7.6% for estradiol). Free testosterone was calculated using the Vermeulen formula assuming a constant albumin concentration of 43 g/L.^29^

### 2.3 Covariates

We used knowledge from previous studies and Directed Acyclic Graphs^30^ for a priori identification of potential confounders (Figure S1). We included parental couple fecundity (measured by time to pregnancy and use of medically assisted reproduction), first trimester highest socioeconomic status of the parents, and maternal first trimester smoking, pre‐pregnancy body mass index, and age at delivery. Information on age at delivery was obtained from the Danish Medical Birth Registry, and the remaining covariates were obtained from the first DNBC interview. Socioeconomic status was defined according to occupation and level of education derived from the Danish International Standard Class of Occupation and Education codes (ISCO‐88 and ISCED).

### 2.4 Statistical analyses

Maternal intake of total folate in midpregnancy was categorized into quintiles (lowest quintile: 105–299 μg/day, second quintile: 300–363 μg/day, third quintile: 364–450 μg/day, fourth quintile: 451–814 μg/day, highest quintile: 815–1718 μg/day). We estimated relative percentage differences with 95% confidence intervals (CI) for all markers of male fecundity using multivariable negative binomial regression models (STATA's ‐nbreg‐ package) according to maternal intake of total folate with the highest quintile as reference. Then, to avoid introducing arbitrary cutoffs and to examine potential dose‐dependency, we fitted the linear association per standard deviation (SD) decrease in total folate (SD: 318 μg/day). Lastly, we fitted a restricted cubic spline with three knots (at 10th percentile: 258 μg/day, 50th percentile: 401 μg/day, 90th percentile: 1058 μg/day) to visualize the association between total folate and the markers of male fecundity.

In addition to the potential confounding factors, all models were fitted with precision variables, that is, variables with an expected strong association with the outcomes, to improve precision. Information on the precision variables was recorded at the clinical visit. We included abstinence time (days), place of semen sample collection (home/clinic), and spillage of semen sample (yes/no) in models examining semen characteristics; however, participants reporting spillage (*n* = 149) were excluded from models that examined volume and total sperm count. Interval from ejaculation to analysis (minutes) was furthermore included in models examining motility. To ensure optimal model fit, we modeled the percentage of non‐progressive + immotile spermatozoa in the statistical analyses of sperm motility instead of percentage of progressive motile sperm. Therefore, positive estimates represent a relatively lower percentage of progressive motile spermatozoa and negative estimates represent a relatively higher percentage of motile spermatozoa. We included abstinence time in models examining testes volume, and we included time of the day at blood sampling in models examining reproductive hormone levels. We modeled all continuous covariates as second‐order polynomials to allow for non‐linearity.

We fitted all models with selection weights based on early pregnancy supplement intake, the potential confounders, and region of the invited men to consider potential selection bias because of non‐participation^31^ and robust standard errors to account for the use of the weights and the clustering of siblings.

We checked all models by comparing the observed distributions against the model‐based distributions in *Q*–*Q* plots and by plotting standardized deviance residuals against the model‐based predictions. The model fit was satisfactory. We presented all percentiles including minimum and maximum values as pseudo percentiles that were calculated as the mean of the five values nearest to the actual percentile because of local regulations (GDPR, Regulation (EU), 2016/679 of 25 May 2018). We conducted data management and statistical analyses in STATA 17.0 (StataCorp, College Station, TX).

### 2.5 Subanalyses

Several subanalyses were conducted. We further adjusted for maternal diet quality by the use of a healthy eating index to consider some maternal healthy lifestyle. We also examined the independent effect of folate from diet and folic acid from supplements separately, to explore any potential separate effect. Lastly, we investigated intake of supplements during early pregnancy, as early pregnancy may be considered the most important exposure window with regard to male fecundity.^32^ The analyses are presented in the Supporting Information section.

## 3 RESULTS

Median midpregnancy intake of total folate in the 787 mothers of the sons in the study population was 401 μg/day (range 105–1718 μg/day). Non‐participants had a similar intake of total folate (median intake: 414 μg/day). Early pregnancy intake of supplements containing folic acid only reported in the enrolment form was also similar in participants (23% were users) and non‐participants (22% were users).

Mothers with the lowest intake of total folate were more likely to be overweight or obese and more likely to report first trimester smoking compared to mothers with a higher intake. Mothers, who had the highest intake of total folate, were more likely to report a time to pregnancy of more than 12 months or medically assisted reproduction than mothers with a lower intake (Table 1). Overall, smoking and lower socioeconomic status of the parents was associated with impaired markers of male fecundity, whereas the other identified potential confounding factors were not consistent associated with markers of male fecundity. Sperm concentration and total sperm count were slightly lower in sons exposed to the lowest quintile of total folate in midpregnancy (Table 2).

**TABLE 1:** Baseline characteristics

	Maternal total folate intake in μg/day of dietary folate equivalents (DFEs)											
	Lowest quintile		Second quintile		Third quintile		Fourth quintile		Highest quintile		Missing	
	No.	%	No.	%	No.	%	No.	%	No.	%	No.	%
	157	20	158	20	157	20	15	20	157	20		
Total DFE: p50 (range)	258 (105–299)		328 (300–363)		401 (364–450)		535 (451–814)		1058 (815–1718)			
Folate from diet: p50 (range)	259 (105–299)		328 (205–362)		399 (197–450)		473 (181–696)		380 (168–1107)			
Folic acid from supplements: p50 (range)	0 (0–3)		0 (0–14)		0 (0–109)		0 (0–298)		400 (100–720)			
Baseline characteristics												
Highest social class of parents											0	0
High‐grade professional	53	34	55	35	51	32	52	33	55	35		
Low‐grade professional	47	30	52	33	59	38	58	37	45	29		
Skilled or unskilled worker	51	25	40	25	41	26	42	27	51	32		
Student/economically inactive	6	4	11	7	6	4	6	4	6	4		
Maternal age at delivery (years (SD))	30.5 (3.9)		31.0 (4.7)		31.5 (3.8)		31.0 (4.3)		31.0 (3.9)		<5^b^	1
Maternal pre‐pregnancy BMI (kg/m^2^)											17	2
<18.5	8	5	7	4	9	6	12	8	9	6		
18.5–24.9	<102^b^	<65	119	75	123	78	<118^b^	<75	<116^b^	<74		
25–29.9	32	20	23	15	18	11	19	12	27	17		
>30	15	10	<5^b^	<3	<5^b^	<3	9	6	5	3		
Maternal smoking first trimester (cigarettes/day)											0	0
0	116	74	124	78	125	79	125	79	122	78		
1–10	31	20	>29^b^	>18	>27^b^	>17	27	17	28	18		
>10	10	7	<5^b^	<3	<5^b^	<3	6	4	7	4		
TTP incl. unplanned pregnancy and MAR											5	1
Unplanned pregnancy	21	13	26	16	16	10	33	21	20	13		
TTP 0–5 months	<100^b^	<64	<100^b^	<64	106	68	<97^b^	<62	85	54		
TTP 6–12 months	18	11	15	9	15	20	10	6	22	14		
TTP > 12 months or MAR	18	11	17	11	20	13	18	11	30	19		
Precision variables												
Abstinence time (days [SD])	2.3 (1.6)		2.4 (1.3)		2.5 (1.4)		2.3 (1.2)		2.4 (1.9)		<5^b^	1
Place of semen sample collection											7	1
At home	29	18	36	23	19	12	15	9	<5^b^	<3		
In the clinic	<128^b^	<82	<122^b^	<77	<138^b^	<88	<143^b^	<91	<153^b^	<97		
Spillage											5	1
Yes	30	19	25	16	30	19	32	20	26	17		
No	<127^b^	<81	133	84	<127^b^	<81	<126^b^	<80	<131^b^	<83		
Interval ejaculation—analysis (min [SD])	51.0 (22.5)		54.0 (23.5)		50.0 (20.0)		48.5 (15.5)		48.5 (11.0)		6	1
Time at blood sample collection											6	1
Morning <12 pm	53	34	60	38	58	37	57	36	47	30		
Afternoon 12–18 pm	<87^b^	<55	77	49	<82^b^	<52	<89^b^	<56	<92^b^	<59		
Evening > 18 pm	17	11	21	13	17	11	12	8	18	11		

*Note*: Distribution of covariates according to quintiles of maternal intake of folate in μg/day of DFEs^a^ in 787 participants from the FEPOS cohort, Denmark, 1998–2019. Numbers in table reported as *n* (%), mean (SD), or p50 (pseudo range). Percentage may not add up because of rounding to the nearest number.

Abbreviations: BMI, body mass index; DFE, dietary folate equivalents; FEPOS, Fetal Programming of Semen quality; MAR, medically assisted reproduction; p50, pseudo median; SD, standard deviation; TTP, time to pregnancy.

^a^ Total folate intake expressed as DFEs calculated as DFE = folate from food in μg/day + folic acid from vitamins in μg/day × 1.7.

^b^ Because of local data regulations, it is not allowed to report numbers smaller than five. Therefore, some numbers in the table have been rounded up or down to mask the numbers smaller than five.

**TABLE 2:** **Markers of male fecundity** **^a^** according to quintiles of maternal intake of folate in μg/day of dietary folate equivalents (DFEs)^b^ in 787 participants from the Fetal Programming of Semen quality (FEPOS) cohort, Denmark, 1998–2019

	Maternal total folate intake in μg/day of dietary folate equivalents (DFEs)											
	Lowest quintile		Second quintile		Third quintile		Fourth quintile		Highest quintile		Missing	
	No.	%	No.	%	No.	%	No.	%	No.	%	No.	%
	157	20	158	20	157	20	158	20	157	20		
Semen quality characteristics												
Volume (ml)^d^	2.5 (1.7–3.4)		2.8 (2.0–3.6)		2.7 (1.8–3.8)		3.0 (2.1–3.9)		2.7 (2.0–3.7)		149	19
Concentration (mill/ml)	35 (16–68)		40 (20–67)		39 (23–67)		37 (17–66)		41 (18–73)		<5^c^	<1
Total sperm count (mill)^d^	88 (38–174)		120 (46–191)		96 (55–195)		111 (52–207)		101 (46–228)		149	19
Motility (PR%)^e^	63 (54–74)		65 (55–74)		67 (54–76)		62 (50–74)		57 (48–69)		19	2
Morphology (% normal)^e^	6 (3–10)		6 (2–11)		7 (4–10)		6 (4–10)		6 (3–10)		24	3
DFI (%)	9 (6–12)		10 (7–14)		10 (7–14)		9 (6–13)		9 (6–12)		52	7
HDS (%)	10 (7–13)		10 (7–13)		9 (7–12)		9 (7–12)		9 (6–13)		52	7
Testicular volume (ml)	15 (11–20)		15 (12–20)		15 (12–20)		15 (11–20)		16 (12–20)		<5^c^	<1
Reproductive hormones												
Testosterone (nmol/L)	18 (15–22)		18 (15–22)		18 (15–22)		18 (15–22)		18 (15–22)		6	1
Estradiol (pmol/L)	52 (34–71)		55 (36–76)		52 (34–76)		52 (32–75)		48 (30–67)		6	1
SHBG (nmol/L)	32 (25–40)		31 (24–40)		32 (25–42)		34 (26–42)		33 (25–41)		7	1
FSH (IU/L)	3.2 (2.4–4.6)		4.1 (2.8–5.0)		3.3 (2.4–5.2)		3.6 (2.5–5.4)		3.3 (2.3–5.2)		7	1
LH (IU/L)	5.1 (4.0–6.7)		5.2 (4.2–6.9)		5.0 (4.1–6.6)		5.4 (4.1–6.7)		5.0 (3.7–6.5)		7	1
Free testosterone (nmol/L)	0.4 (0.3–0.5)		0.4 (0.3–0.5)		0.4 (0.3–0.5)		0.4 (0.3–0.5)		0.4 (0.3–0.5)		7	1

Abbreviations: DFEs, dietary folate equivalents; DFI, DNA fragmentation index; FSH, follicle‐stimulating hormone; HDS, high DNA stainability; IQR, pseudo intra quartile range; LH, luteinizing hormone; p50, 50th pseudo percentile; PR, progressive motility.

^a^ Reported as 50th percentile (IQR). All percentiles are pseudo percentiles calculated from the average of five values.

^b^ Total folate intake expressed as DFEs calculated as DFE = folate from food in μg/day + folic acid from vitamins in μg/day × 1.7.

^c^ Because of local data regulations, it is not allowed to report numbers smaller than five. Therefore, some numbers in the table have been rounded up or down to mask the numbers smaller than five.

^d^ Excluding samples from participants reporting spillage.

^e^ Excluding azoospermia samples.

Prenatal exposure to low maternal intake of total folate in midpregnancy was associated with a lower total sperm count of −5% (95% CI: −11%; 2%), a lower proportion of non‐progressive and immotile spermatozoa of −5% (95% CI: −3%; −8%), and lower testes volume of −4% (95% CI: −6%; −2%) per SD decrease in total folate (Table 3). The association with lower sperm count was most pronounced in young men of mothers having the lowest intake of total folate (lowest quintile of total folate relative to highest quintile was −18% [95% CI: −35%; 2%]). The spline plots (Figure 2) overall confirmed these results; lower maternal intake of total folate was associated with a lower proportion of non‐progressive and immotile spermatozoa and lower testes volume in a dose‐dependent manner (both *p*‐values <0.01), and that men of mothers having the lowest intake of total folate had a tendency toward lower sperm counts (*p* = 0.2). Maternal intake of total folate was also associated with DFI in an inverse U‐shaped manner; hence, we observed associations with higher DFI in the second, third, and fourth quintile relative to exposure to the fifth quintile. No consistent associations with reproductive hormone levels were observed (Table 3 and Figure 2).

**TABLE 3:** Main analysis

	*n* ^d^		Crude	Adjusted (95% CI)
Semen characteristics^e^				
Volume (ml)^f^	615	Lowest	−8%	−9% (−19; 4)
		Second	0%	−3% (−13; 8)
		Third	2%	−2% (−13; 10)
		Fourth	7%	6% (−5; 17)
		Highest	2.9 ml	Reference
Continuous (per SD decrease)			−1%	−2% (−6; 1)
Concentration (mill/ml)	752	Lowest	−11%	−2% (−9; 19)
		Second	−6%	5% (−13; 28)
		Third	−7%	4% (−13; 24)
		Fourth	−8%	4% (−14; 26)
		Highest	53 mill/mL	Reference
Continuous (per SD decrease)			−3%	1% (−5; 7)
Total sperm count (mill)^f^	615	Lowest	−18%	−18% (−35; 2)
		Second	−5%	1% (−19; 26)
		Third	−8%	−8% (−25; 13)
		Fourth	0%	4% (−15; 28)
		Highest	154 mill	Reference
Continuous (per SD decrease)			−6%	−5% (−11; 2)
Motility (modeled as NP + IM%)^g^	734	Lowest	−14%	−13% (−21; −5)
		Second	−16%	−14% (−22; −6)
		Third	−16%	−14% (−22; −6)
		Fourth	−10%	−9% (−16; 0)
		Highest	43%	Reference
Continuous (per SD decrease)			−6%	−5% (−8; −3)
Morphology (% normal)	732	Lowest	3%	6% (−11; 26)
		Second	4%	12% (−7; 33)
		Third	3%	9% (−7; 28)
		Fourth	0%	1% (−14; 18)
		Highest	6.7%	Reference
Continuous (per SD decrease)			1%	3% (−2; 9)
DFI (%)	703	Lowest	5%	1% (−11; 16)
		Second	14%	16% (1; 32)
		Third	16%	17% (3; 33)
		Fourth	10%	19% (2; 38)
		Highest	9.7%	Reference
Continuous (1‐SD)			3%	3% (−1; 7)
HDS (%)	703	Lowest	8%	6% (−7; 20)
		Second	10%	5% (−9; 20)
		Third	2%	2% (−10; 15)
		Fourth	3%	2% (−11; 17)
		Highest	9.8%	Reference
Continuous (per SD decrease)			2%	1% (−3; 6)
Testicular volume^h^				
Average testicular volume (ml)	758	Lowest	−9%	−10% (−16; −3)
		Second	8%	−11% (−17; −5)
		Third	4%	−7% (−13; 1)
		Fourth	6%	−8% (−15; −1)
		Highest	17 ml	Reference
Continuous (per SD decrease)			−3%	−4% (−6; −2)
Reproductive hormones^i^				
Testosterone (nmol/L)	758	Lowest	−1%	−2% (−8; 5)
		Second	−2%	−4% (−10; 3)
		Third	−1%	−3% (−10; 4)
		Fourth	−1%	−3% (−9; 4)
		Highest	18.8 nmol/L	Reference
Continuous (per SD decrease)			−1%	−1% (−3; 1)
Estradiol (pmol/L)	758	Lowest	7%	6% (−6; 20)
		Second	12%	13% (0; 27)
		Third	8%	10% (−2; 24)
		Fourth	6%	5% (−8; 19)
		Highest	51.5 pmol/L	Reference
Continuous (per SD decrease)			3%	3% (0; 7)
SHBG (nmol/L)	757	Lowest	−4%	−4% (−12; 4)
		Second	−3%	−6% (−14; 2)
		Third	1%	−3% (−11; 7)
		Fourth	1%	−1% (−9; 8)
		Highest	34.8 nmol/L	Reference
Continuous (per SD decrease)			−2%	−2% (−5; 0)
FSH (IU/L)	757	Lowest	−6%	−3% (−16; 12)
		Second	7%	8% (−6; 24)
		Third	0%	−4% (−18; 11)
		Fourth	8%	9% (−11; 34)
		Highest	4.1 IU/L	Reference
Continuous (per SD decrease)			0%	0% (−4; 5)
LH (IU/L)	757	Lowest	3%	2% (−7; 11)
		Second	9%	10% (1; 20)
		Third	6%	5% (−4; 15)
		Fourth	12%	10% (−2; 24)
		Highest	5.2 IU/L	Reference
Continuous (per SD decrease)			3%	2% (0; 5)
Free testosterone (nmol/L)	757	Lowest	0%	1% (−7; 6)
		Second	0%	−1% (−7; 5)
		Third	−2%	−3% (−9; 3)
		Fourth	−1%	−3% (−10; 4)
		Highest	0.4 nmol/L	Reference
Continuous (per SD decrease)			0%	−1% (−3; 2)

*Note*: Crude and adjusted^a^ (95% CI) relative percentage differences in markers of male fecundity according to prenatal exposure to folate in μg/day of DFEs^b^ and per standard deviation decrease^c^ in 787 participants from the FEPOS cohort, Denmark, 1998–2019.

Abbreviations: BMI, body mass index; CI, confidence intervals; DFE, dietary folate equivalents; DFI, DNA fragmentation index; FEPOS, Fetal Programming of Semen quality; FSH, Follicle‐stimulating hormone; HDS, High DNA stainability; IM, Immotile; LH, Luteinizing hormone; MAR, medically assisted reproduction; NP, non‐progressive motility; SD, standard deviation; SHBG, sex‐hormone binding globulin; TTP, time to pregnancy.

^a^ Adjusted for maternal age at delivery, highest parental social class, maternal 1. Trimester smoking, maternal pre‐pregnancy BMI, TTP including MAR.

^b^ Total folate intake expressed as DFEs calculated as DFE = folate from food in μg/day + folic acid from vitamins in μg/day × 1.7.

^c^ SD = 318 μg/day.

^d^ The numbers are from the adjusted model and vary because of the exclusion of azoospermia semen samples and because of potential missingness on covariates.

^e^ Further adjusted for abstinence time, spillage and place of semen sample collection.

^f^ Excluding samples with spillage.

^g^ Further adjusted for interval from ejaculation to analysis of motility. Estimates represent the relative difference in the proportion of non‐progressive and immotile spermatozoa. Therefore, positive estimates should be interpreted as a relatively lower progressive motility and vice versa.

^h^ Further adjusted for abstinence time.

^i^ Further adjusted for time of blood sample drawing.

FIGURE 2Spline plots of the association between prenatal exposure to maternal intake of total folate in midpregnancy and markers of male fecundity. Restricted cubic spline plots (three knots at 10th percentile: 258 μg/day, 50th percentile: 401 μg/day, 90th percentile: 1058 μg/day) of markers of male fecundity according to prenatal exposure to maternal intake of total folate in dietary equivalents (DFEs) in μg/day assessed in midpregnancy (solid lines) with 95% confidence intervals (dotted lines). The estimated measures are presented for a reference son, whose parents had a waiting time to pregnancy of 1–5 months, and whose parents’ highest social class was a high‐grade professional, whose mother was 30 years at the delivery, was normal weight, and was a non‐smoker. The reference son had an abstinence time of 2.5 days, delivered his semen sample at the clinic, did not report any spillage of the semen sample, had his motility assessment performed 30 min after ejaculation, and had blood drawn for assessment of reproductive hormone levels from 12 to 18 pm Abbreviations: FSH, follicle‐stimulating hormone; IM, immotile motility; LH, luteinizing hormone; NP, non‐progressive motility; SHBG, sex‐hormone binding globulin.

**Figure:**
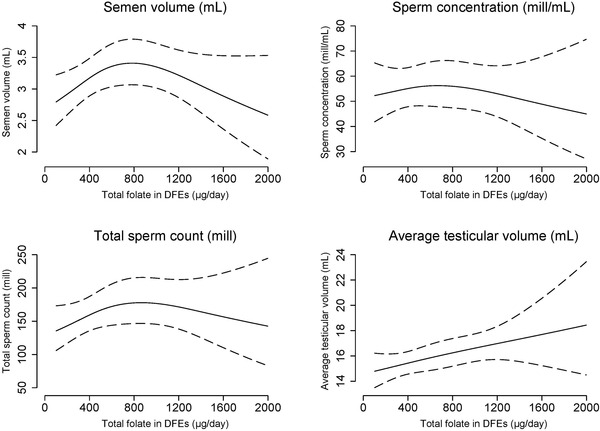

**Figure:**
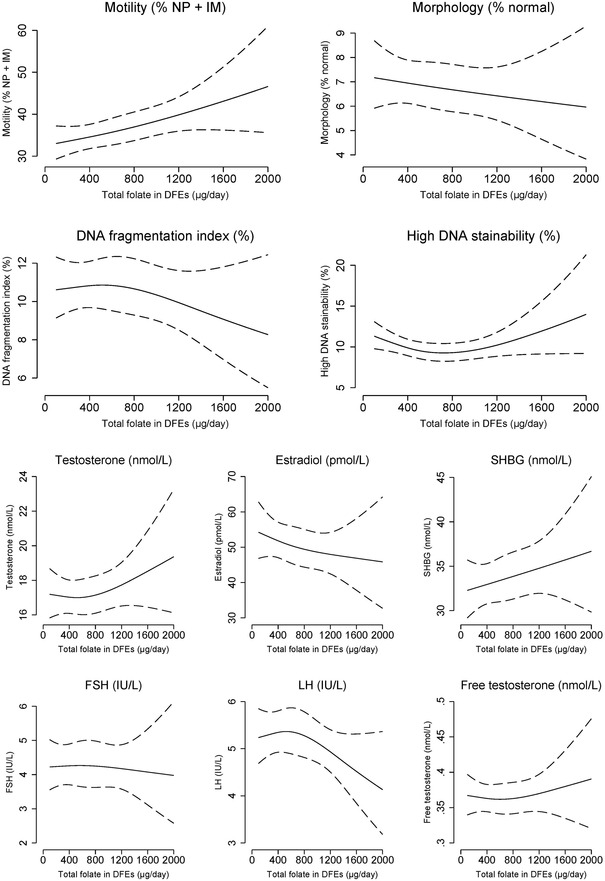

### 3.1 Subanalyses

The associations were overall similar when we further adjusted for a healthy eating index (Table S1). The associations with total sperm count, motility, and testes volume remained, when examining midpregnancy folic acid from supplements alone (Table S2), whereas the dose‐dependent associations with a lower proportion of non‐progressive and immotile spermatozoa were not observed, when investigating folate from diet alone (Table S3). Most baseline characteristics differed according to early pregnancy intake of supplements (Table S4), whereas crude measures of male fecundity did not (Table S5). Early pregnancy exposures to folic acid from supplements were not associated with markers of male fecundity (Table S6).

## 4 DISCUSSION

### 4.1 Key results

In young men, lower maternal intake of total folate in midpregnancy was associated with lower total sperm count, a lower proportion of non‐progressive and immotile spermatozoa, and lower testes volume.

### 4.2 Strengths and limitations

The major strengths of this study included the longitudinal design, that is, information on maternal intake of total folate was obtained in pregnancy, and the use of quality controlled and valid measures of male fecundity was measured around 19 years later. We adjusted for a number of potential confounding factors in addition to precision variables. Although the participation rate was low (19%), potential selection bias because of non‐participation in the FEPOS cohort is not considered to be of concern.^33^ In addition, participation was unrelated to maternal intake of total folate, and we further considered potential selection bias because of loss‐to‐follow‐up by applying inverse probability weights.^31^ We therefore consider the risk of selection bias limited.

Information on total folate intake was obtained from an FFQ. This implies a risk of information bias because of errors in maternal self‐reporting of intake of diet and supplements, and in the calculation of folate content. However, the use of the DNBC FFQ to assess total folate has been found to be valid, because folate intake assessed as DFEs and erythrocyte folic acid, a biomarker of long‐term total folate intake, was correlated (Spearman correlation coefficient was 0.55 [*p* < 0.0001]).^34^ The measurement errors are further expected to be non‐differential and independent on the markers of male fecundity and therefore not likely to explain the observed associations.

Markers of male fecundity may also be subject to measurement errors. However, this is also most likely non‐differential and independent of maternal intake of total folate. The measurements of semen characteristics and reproductive hormone levels were performed by skilled laboratory technicians blinded to the participants’ exposure status, and were continuously quality‐controlled and met given standards for semen analyses.^21^ The within individual variation in semen quality may not introduce any systematic errors.^35^ The participants have probably not under or overestimated their testes volume according to the exposure status.^28^ Finally, we considered the daily fluctuations in reproductive hormone levels by adjusting for time a day of blood draw in the analyses.

Confounding is an inherent risk in observational studies. Although we adjusted for a number of potential confounding factors, residual confounding may still affect the observed results. However, further adjustment for the quality of maternal diet in midpregnancy, which may capture some unmeasured maternal healthy lifestyle, overall did not change the results, suggesting limited confounding by maternal diet quality.

### 4.3 Interpretation

Prenatal exposure to folate may epigenetically program the fetus and affect long‐term health, including reproductive health. We found that low maternal intake of total folate was associated with lower testes volume, which may be explained by folate's crucial role in the biosynthesis of nucleotides, the rate of cell divisions, gene expression through aberrant DNA methylations, and, hence, growth.^13^ The current understanding of folate's role in fetal programming is not fully elucidated; therefore, the finding of a lower proportion of non‐progressive and immotile spermatozoa and the apparent inverse U‐shaped association for DFI was surprising and may represent chance findings.

Although we hypothesized that maternal folate may affect male fecundity via changes in DNA methylations, we were unable to specifically assess this in our study. Folate displays anti‐oxidative properties,^36^ and it is also possible that maternal folate intake may protect against harmful effects of environmental exposures on markers of male fecundity.^37^ For example, a study in rats showed that the harmful effects of prenatal exposure to persistent organic pollutants on male reproductive function were mitigated by maternal folic acid supplement during pregnancy.^37^

Concern has been raised that high exposure to folic acid, not naturally occurring folate from the diet, may be associated with some adverse effects in the offspring, such as hypersensitivity outcomes.^38^ Therefore, we further examined the separate effect of folic acid from supplements and folate from the diet on the markers of male fecundity. Interestingly, we did not observe associations with motility, when only examining maternal intake of folate from diet, whereas we found associations between a higher maternal intake of folic acid from supplements and a higher proportion of non‐progressive and immotile spermatozoa (Supporting Information). Whether our findings suggest a potential adverse effect of folic acid on sperm motility should be further investigated.

Only one previous study has investigated the association between maternal folate intake and markers of male fecundity and found no associations.^19^ In the study, the pregnant women provided information on intake of folic acid at 36 gestational weeks. More than half of the pregnant women (53%) had missing information on intake of folic acid. Moreover, they provided no information on dose, frequency or duration of intake of folic acid, or intake of folate from the diet. Similar to our subanalysis of early pregnancy intake of supplementation (Supporting Information), these exposure groups are likely very crude measures of actual fetal exposure to folic acid. This offers a potential explanation of the null findings.^19^

The use of a reliable biomarker of total folate, such as erythrocyte‐folate, should be considered in future studies. Moreover, to promote causal interpretation, Mendelian randomization studies, using genetic information to predict circulating concentrations of total folate exposure, would strengthen the confidence in the results by reducing the risk of potential exposure‐outcome confounding.^39^

Maternal intake of folic acid supplements during second and third pregnancy trimesters has been associated with altered DNA methylation patterns that persist into adulthood.^14^ However, early pregnancy, where the reproductive organs develop, may constitute a crucial exposure window for low folate exposure with regard to markers of male fecundity.^32^ We investigated this in a subanalysis only, as we did not have any information on dose or duration or frequency of intake and the exposure categories were therefore very crude measures of actual fetal exposure to folic acid. We found no associations between maternal intake of dietary supplements and markers of fecundity (Supporting Information).

A dietary supplementation of 400 μg folic acid per day prior to conception and in early pregnancy is recommended to reduce the risk of neural tube defects.^40^ In our study population, 15% (*n* = 120) of the women had an intake from supplements of 400 μg/day, when they were asked in mid pregnancy; however, we had no information on compliance in the periconceptional period. During pregnancy, intake of total folate should be at least 500–600 μg/day from diet and from supplements.^11^, ^15^, ^41^ Only 267 mothers (34%) in our study population had an intake of total folate of more than 500 μg/day in mid pregnancy. Despite this low compliance, food fortification with folic acid is not mandatory in Denmark or in most of the European Union, as opposed to countries such as Canada, USA, and UK.^38^ Because of differences in bioavailability of folic acid from supplements and fortified food and folate from diet as well as differences in dietary sources providing folic acid from fortified foods (flour, rice, pasta, and cereal products) and folate from diet (leafy green vegetables, fruit and fruit juice, peas, and beans),^11^ our results may generalize to other populations, where food fortification is not mandatory.

In conclusion, maternal intake of total folate may influence male reproductive health, as we observed associations with total sperm count, motility, and testes volume. The results were ambiguous, as we found signals of both potential lower fecundity in some markers and higher fecundity in other markers of male fecundity. This warrants further investigation. Regardless, pregnant women should still follow the current recommendations on folate intake, because of the well‐established benefits for children's health following maternal intake of sufficient folate in the periconceptional period.

## AUTHOR CONTRIBUTIONS

Birgit Bjerre Høyer and Cecilia Høst Ramlau‐Hansen planned and acquired the funding for the study. Anne Gaml‐Sørensen performed data management and designed the analysis strategy, performed the statistical analyses, and wrote the first draft in close collaboration with Nis Brix, Gunnar Toft, Tine Brink Henriksen, and Cecilia Høst Ramlau‐Hansen. The funding for FEPOS was acquired by Sandra Søgaard Tøttenborg and Jens Peter Ellekilde Bonde. The data collection in FEPOS was planned and headed by Gunnar Toft, Birgit Bjerre Høyer, Sandra Søgaard Tøttenborg, Karin Sørig Hougaard, Jens Peter Ellekilde Bonde, and Cecilia Høst Ramlau‐Hansen. All authors had full access to all the data in the study, all authors interpreted the data, revised the manuscript critically, approved, and accepted responsibility of the final manuscript.

## CONFLICTS OF INTEREST

The authors have no conflicts of interest to disclose.

## Supporting information

Supporting Information

## Data Availability

The dataset analyzed in the study is not publicly available because of national data security legislation on sensitive personal data. Researchers may apply for access to data from the DNBC. Please see http://www.dnbc.dk/data‐available for additional information.

## References

[1] Levine H , Jørgensen N , Martino‐Andrade A , et al. Temporal trends in sperm count: a systematic review and meta‐regression analysis. Hum Reprod Update. 2017;23(6):646‐659. doi:10.1093/humupd/dmx022 28981654 PMC6455044

[2] Skakkebaek NE , Rajpert‐De Meyts E , Buck Louis GM , et al. Male reproductive disorders and fertility trends: influences of environment and genetic susceptibility. Physiol Rev. 2016;96(1):55‐97. Epub 2015/11/20. doi:10.1152/physrev.00017.2015 26582516 PMC4698396

[3] Virtanen HE , Jørgensen N , Toppari J . Semen quality in the 21(st) century. Nat Rev Urol. 2017;14(2):120‐130. Epub 2017/01/05. doi:10.1038/nrurol.2016.261 28050014

[4] Jørgensen N , Joensen UN , Jensen TK , et al. Human semen quality in the new millennium: a prospective cross‐sectional population‐based study of 4867 men. BMJ Open. 2012;2(4):e000990. doi:10.1136/bmjopen-2012-000990 PMC3391374 22761286

[5] Bonde JP , Ernst E , Jensen TK , et al. Relation between semen quality and fertility: a population‐based study of 430 first‐pregnancy planners. Lancet (London, England). 1998;352(9135):1172‐1177. Epub 1998/10/20. doi:10.1016/s0140-6736(97)10514-1 9777833

[6] Trasande L , Zoeller RT , Hass U , et al. Estimating burden and disease costs of exposure to endocrine‐disrupting chemicals in the European union. J Clin Endocrinol Metab. 2015;100(4):1245‐1255. Epub 2015/03/06. doi:10.1210/jc.2014-4324 25742516 PMC4399291

[7] Skakkebæk NE , Rajpert‐De Meyts E , Main KM . Testicular dysgenesis syndrome: an increasingly common developmental disorder with environmental aspects: opinion. Hum Reprod. 2001;16(5):972‐978. doi:10.1093/humrep/16.5.972 11331648

[8] Sharpe RM , Skakkebaek NE . Are oestrogens involved in falling sperm counts and disorders of the male reproductive tract? Lancet (London, England). 1993;341(8857):1392‐1395. Epub 1993/05/29. doi:10.1016/0140-6736(93)90953-e 8098802

[9] Sánchez‐Garrido MA , García‐Galiano D , Tena‐Sempere M . Early programming of reproductive health and fertility: novel neuroendocrine mechanisms and implications in reproductive medicine. Hum Reprod Update. 2022;28(3):346‐375. Epub 2022/02/22. doi:10.1093/humupd/dmac005 35187579 PMC9071071

[10] Crider KS , Yang TP , Berry RJ , Bailey LB . Folate and DNA methylation: a review of molecular mechanisms and the evidence for folate's role. Adv Nutr (Bethesda, MD). 2012;3(1):21‐38. doi:10.3945/an.111.000992 PMC3262611 22332098

[11] Standing Committee on the Scientific Evaluation of Dietary Reference Intakes and its Panel on Folate OBV, and Choline. The National Academies Collection: Reports funded by National Institutes of Health. Dietary Reference Intakes for Thiamin, Riboflavin, Niacin, Vitamin B(6), Folate, Vitamin B(12), Pantothenic Acid, Biotin, and Choline. Washington (DC): National Academies Press (US); 1998. Chapter 8. PMID: 23193625. Bookshelf ID: NBK114310 DOI: 10.17226/6015 Found at: https://pubmed.ncbi.nlm.nih.gov/23193625/

[12] Silva C , Keating E , Pinto E . The impact of folic acid supplementation on gestational and long term health: critical temporal windows, benefits and risks. Porto Biomed J. 2017;2(6):315‐332. Epub 2017/11/01. doi:10.1016/j.pbj.2017.05.006 32258789 PMC6806748

[13] McGee M , Bainbridge S , Fontaine‐Bisson B . A crucial role for maternal dietary methyl donor intake in epigenetic programming and fetal growth outcomes. Nutr Rev. 2018;76(6):469‐478. Epub 2018/03/13. doi:10.1093/nutrit/nuy006 29529267

[14] Richmond RC , Sharp GC , Herbert G , et al. The long‐term impact of folic acid in pregnancy on offspring DNA methylation: follow‐up of the Aberdeen Folic Acid Supplementation Trial (AFAST). Int J Epidemiol. 2018;47(3):928‐937. Epub 2018/03/17. doi:10.1093/ije/dyy032 29546377 PMC6005053

[15] Tamura T , Picciano MF . Folate and human reproduction. Am J Clin Nutr. 2006;83(5):993‐1016. Epub 2006/05/11. doi:10.1093/ajcn/83.5.993 16685040

[16] Rotondo JC , Lanzillotti C , Mazziotta C , Tognon M , Martini F . Epigenetics of male infertility: the role of DNA methylation. Front Cell Dev Biol. 2021;9:689624. Epub 2021/08/10. doi:10.3389/fcell.2021.689624 34368137 PMC8339558

[17] Cui X , Jing X , Wu X , et al. DNA methylation in spermatogenesis and male infertility. Exp Ther Med. 2016;12(4):1973‐1979. Epub 2016/10/05. doi:10.3892/etm.2016.3569 27698683 PMC5038464

[18] Rajender S , Avery K , Agarwal A . Epigenetics, spermatogenesis and male infertility. Mutat Res/Rev Mutat Res. 2011;727(3):62‐71. doi:10.1016/j.mrrev.2011.04.002 21540125

[19] Jacobsen K , Ramlau‐Hansen CH , Thulstrup AM , Olsen J , Bonde JP . Maternal folic acid supplement intake and semen quality in Danish sons: a follow‐up study. Fertil Steril. 2011;96(2):295‐298. Epub 2011/06/15. doi:10.1016/j.fertnstert.2011.05.037 21664612

[20] Olsen J , Melbye M , Olsen SF , et al. The Danish National Birth Cohort—its background, structure and aim. Scand J Public Health. 2001;29(4):300‐307. Epub 2002/01/05. PubMed PMID: 11775787. 11775787 10.1177/14034948010290040201

[21] Keglberg Hærvig K , Bonde JP , Ramlau‐Hansen CH , et al. Fetal Programming of Semen quality (FEPOS) cohort—A DNBC male‐offspring cohort. Clin Epidemiol. 2020;12:757‐770. Epub 2020/08/09. doi:10.2147/clep.S242631 32765110 PMC7373412

[22] Olsen SF , Mikkelsen TB , Knudsen VK , et al. Data collected on maternal dietary exposures in the Danish National Birth Cohort. Paediatr Perinat Epidemiol. 2007;21(1):76‐86. doi:10.1111/j.1365-3016.2007.00777.x 17239183

[23] Food data made available by National Food Institute , Technical University of Denmark (frida.fooddata.dk)

[24] Saldanha LG , Dwyer JT , Haggans CJ , Mills JL , Potischman N . Perspective: time to resolve confusion on folate amounts, units, and forms in prenatal supplements. Adv Nutr (Bethesda, MD). 2020;11(4):753‐759. doi:10.1093/advances/nmaa017 PMC7360441 32134106

[25] Menkveld R , Stander FS , Kotze TJ , Kruger TF , van Zyl JA . The evaluation of morphological characteristics of human spermatozoa according to stricter criteria. Hum Reprod. 1990;5(5):586‐592. Epub 1990/07/01. PubMed PMID: 2394790. 2394790 10.1093/oxfordjournals.humrep.a137150

[26] Evenson DP . Sperm chromatin structure assay (SCSA®). Methods Mol Biol. 2013;927:147‐164. Epub 2012/09/21. doi:10.1007/978-1-62703-038-0_14 22992911

[27] World Health Organization . WHO Laboratory Manual for the Examination and Processing of Human Semen. 5th ed. World Health Organization; 2010.

[28] Ramlau‐Hansen CH , Thulstrup AM , Bonde JP , Ernst E . Is self‐measuring of testicular volume by a Prader orchidometer a valid method? Fertil Steril. 2007;87(6):1480‐1482. Epub 2007/02/14. doi:10.1016/j.fertnstert.2006.11.032 17296192

[29] Vermeulen A , Verdonck L , Kaufman JM . A critical evaluation of simple methods for the estimation of free testosterone in serum. J Clin Endocrinol Metab. 1999;84(10):3666‐3672. Epub 1999/10/16. doi:10.1210/jcem.84.10.6079 10523012

[30] Greenland S , Pearl J , Robins JM . Causal diagrams for epidemiologic research. Epidemiology. 1999;10(1):37‐48. Epub 1999/01/15. PubMed PMID: 9888278. 9888278

[31] Hernan MA , Hernandez‐Diaz S , Robins JM . A structural approach to selection bias. Epidemiology. 2004;15(5):615‐625. Epub 2004/08/17. PubMed PMID: 15308962. 15308962 10.1097/01.ede.0000135174.63482.43

[32] Sharpe RM . Androgens and the masculinization programming window: human‐rodent differences. Biochem Soc Trans. 2020;48(4):1725‐1735. Epub 2020/08/12. doi:10.1042/bst20200200 32779695 PMC7458408

[33] Gaml‐Sørensen A , Brix N , Tøttenborg SS , et al. Selection bias in a male‐offspring cohort investigating fecundity: is there reason for concern? Hum Reprod. 2022. Epub 2022/11/13. doi:10.1093/humrep/deac241. PubMed PMID:36370427 36370427

[34] Mikkelsen TB , Osler M , Olsen SF . Validity of protein, retinol, folic acid and n‐3 fatty acid intakes estimated from the food‐frequency questionnaire used in the Danish National Birth Cohort. Public Health Nutr. 2006;9(6):771‐778. Epub 2006/08/24. doi:10.1079/phn2005883 16925883

[35] Hart RJ , Doherty DA , McLachlan RI , et al. Testicular function in a birth cohort of young men. Hum Reprod. 2015;30(12):2713‐2724. Epub 2015/09/27. doi:10.1093/humrep/dev244 26409015

[36] Joshi R , Adhikari S , Patro BS , Chattopadhyay S , Mukherjee T . Free radical scavenging behavior of folic acid: evidence for possible antioxidant activity. Free Radic Biol Med. 2001;30(12):1390‐1399. Epub 2001/06/08. doi:10.1016/s0891-5849(01)00543-3 11390184

[37] Lessard M , Herst PM , Charest PL , et al. Prenatal exposure to environmentally‐relevant contaminants perturbs male reproductive parameters across multiple generations that are partially protected by folic acid supplementation. Sci Rep. 2019;9(1):13829. Epub 2019/09/27. doi:10.1038/s41598-019-50060-z 31554827 PMC6761122

[38] Field MS , Stover PJ . Safety of folic acid. Ann NY Acad Sci. 2018;1414(1):59‐71. Epub 2017/11/21. doi:10.1111/nyas.13499 29155442 PMC5849489

[39] Ebrahim S , Davey Smith G . Mendelian randomization: can genetic epidemiology help redress the failures of observational epidemiology? Hum Genet. 2008;123(1):15‐33. Epub 2007/11/27. doi:10.1007/s00439-007-0448-6 18038153

[40] Wilson RD , Wilson RD , Audibert F , et al. Pre‐conception folic acid and multivitamin supplementation for the primary and secondary prevention of neural tube defects and other folic acid‐sensitive congenital anomalies. J Obstet Gynaecol Can. 2015;37(6):534‐552. Epub 2015/09/04. doi:10.1016/s1701-2163(15)30230-9 26334606

[41] Nordic nutrition recommendations 2012 . 1. oplag. ed. Copenhagen: Nordic Council of Ministers; 2013.

