# Supplementary material for: Maternal intake of folate and folic acid during pregnancy and markers of male fecundity: A population‐based cohort study
Source: Andrology. 2022 Dec 26;11(3):537–50. doi: 10.1111/andr.13364 (PMC10947439; doi:10.1111/andr.13364)
Supplement: Supplementary file 1 — Supporting Information [file ANDR-11-537-s001.docx]

**Supporting Information**

**Supporting text 1 page 2**

**Supplementary table S1 page 5**

**Supplementary table S2 page 9**

**Supplementary table S3 page 12**

**Supplementary table S4 page 14**

**Supplementary table S5 page 16**

**Supplementary table S6 page 18**

**Supplementary figure 1 page 20**

**Supporting Information**

*Supporting text 1*

In a subanalysis, we further adjusted for maternal diet quality in mid-pregnancy.^1^ This was assessed as a healthy eating index derived from the FFQ according to adherence to the Danish National Food-Based Dietary Guidelines and the Nordic Nutrition Recommendations. Eight components were generated, for which a score between zero and 10 was assigned according to a high intake of fruits, vegetables, dietary fibers and fish, and a low intake of red meat, saturated fatty acids, sodium, sugar sweetened beverages and added sugar.^1^ Hence, a high sum of the eight scores was compatible with an overall healthier diet. This information was available for a subset of mothers (n = 344) only, and collinearity was found between healthy eating index and DFEs (Pearson correlations coefficient: 0.16 (95% CI: 0.11; 0.21)). Therefore, this variable was not included in the main analysis. We also repeated the main analysis within this smaller population without adjusting for maternal healthy eating index to be able to explore potential selection bias due to missing information of the healthy eating index.

Next, we examined the association between folate from diet and folic acid from supplements and measures of male fecundity separately, to explore any potential distinct effect of folate from diet or folic acid from supplements, respectively. This was done, since concerns has been raised that high exposure to folic acid, not naturally occurring folate from the diet, may also be associated with some adverse effects in the offspring.^2^ We examined folate from diet in quintiles (lowest quintile: 25 -276 µg/day, second quintile: 277-327 µg/day, third quintile: 328-374 µg/day, fourth quintile: 375-446 µg/day, highest quintile (reference): 447-1576 µg/day) and per SD decrease (SD = 123 µg/day). The potential effect of folic acid from mid-pregnancy supplements on markers of male fecundity was examined in categories (0 µg/day, 1 – 399 µg/day, and ≥ 400 µg/day (reference)). We adjusted the analyses for intake of folic acid from mid-pregnancy supplements and for folate from diet, respectively.

We considered early pregnancy an important exposure window with regard to male fecundity,^3^ and explored this in another subanalysis. The pregnant women provided the product name, if she had an intake of any supplements at enrolment, when she was approximately eight weeks pregnant. Based on the folic acid content in the reported supplement, we derived a categorised variable for intake during early pregnancy (no supplement, supplement without folic acid, multivitamin also with folic acid, and supplement only with folic acid).^4^ The women were assigned the category reflecting the highest amount of folic acid, if she reported intake of multiple supplements. Since the women did not report frequency, dose or duration of intake as opposed to the information from the mid pregnancy FFQ, this was investigated in a subanalysis only.

*Results*

The results from the main analysis restricted to the smaller subpopulation with information on the healthy eating index were attenuated slightly compared to the results from the main analysis on the full study population (Supplementary Table 1), suggesting only limited selection bias. Within this smaller subpopulation, the results were essentially similar, with and without further adjustment for the healthy eating index, suggesting limited confounding by maternal diet quality (Supplementary Table 1). When examining folate from diet alone, we still found associations with lower sperm count (-4% (95% CI: -11%; 4%) and lower testes volume (-2% (95% CI: -4%; 0%)), but not with a lower proportion of non-progressive and immotile spermatozoa (0% (95% CI: -3%; 2%)) per SD decrease in folate from diet (Supplementary Table 2). When examining mid-pregnancy folic acid from supplements, the associations with total sperm count, motility and testes volume remained (Supplementary Table 3). Most baseline characteristics differed according to early pregnancy intake of supplements (Supplementary Table 4), while crude measures of male fecundity did not (Supplementary Table 5). We found no associations between early pregnancy intake of supplements and male fecundity (Supplementary Table 6).

*References*

1. Bjerregaard AA, Halldorsson TI, Tetens I, Olsen SF. Mother's dietary quality during pregnancy and offspring's dietary quality in adolescence: Follow-up from a national birth cohort study of 19,582 mother-offspring pairs. PLoS medicine. 2019;16(9):e1002911. Epub 2019/09/13. doi: 10.1371/journal.pmed.1002911. PubMed PMID: 31513597; PubMed Central PMCID: PMCPMC6742222.

2. Field MS, Stover PJ. Safety of folic acid. Ann N Y Acad Sci. 2018;1414(1):59-71. Epub 2017/11/21. doi: 10.1111/nyas.13499. PubMed PMID: 29155442; PubMed Central PMCID: PMCPMC5849489.

3. Sharpe RM. Androgens and the masculinization programming window: human-rodent differences. Biochem Soc Trans. 2020;48(4):1725-1735. Epub 2020/08/12. doi: 10.1042/bst20200200. PubMed PMID: 32779695; PubMed Central PMCID: PMCPMC7458408.

4. Olsen SF, Mikkelsen TB, Knudsen VK, Orozova-Bekkevold I, Halldórsson TI, Strøm M, Østerdal ML. Data collected on maternal dietary exposures in the Danish National Birth Cohort. Paediatric and perinatal epidemiology. 2007;21(1):76-86. doi: 10.1111/j.1365-3016.2007.00777.x.

| **Supplementary Table 1. Sub analysis.** Crude and adjusted^a^ (95% confidence intervals) relative percentage differences in markers of male fecundity according to prenatal exposure to folate in μg/day of dietary folate equivalents (DFEs)^b^ and per standard deviation decrease^c^ in 344 participants from the fetal programming of semen quality (FEPOS) cohort, Denmark, 1998 - 2019. A: Main analysis with restriction to participants with information on healthy eating index but without adjusting for healthy eating index. B: Main analysis further adjusted for maternal healthy eating index. | | | | | | | | | | |
| --- | --- | --- | --- | --- | --- | --- | --- | --- | --- | --- |
|  |  |  |  |  |  |  |  |  |  |  |
|  |  |  |  |  |  |  |  |  |  |  |
|  |  |  |  |  |  |  |  |  |  |  |
|  |  |  |  |  |  |  |  |  |  |  |
|  |  |  |  |  |  |  |  |  |  |  |
|  |  |  |  |  |  |  |  |  |  |  |
|  |  |  |  |  | A: Restriction | | | B: Further adjusting for maternal healthy eating index | | |
|  |  |  |  |  |  |  |  |  |  |  |
|  |  |  |  |  | Crude | Adjusted (95% CI) | | Crude | Adjusted (95% CI) | |
|  |  |  |  |  |  |  |  |  |  |  |
| Semen characteristics^e^ | | n^d^ |  |  |  |  |  |  |  |  |
|  | Volume (ml)^f^ | 265 | lowest |  | -6% | -1% (-21; 23) | | -6% | -1% (-21; 24) | |
|  |  |  | 2nd |  | -4% | -4% (-19; 13) | | -4% | -4% (-19; 13) | |
|  |  |  | 3rd |  | 8% | 14% (-2; 33) | | 8% | 14% (-2; 33) | |
|  |  |  | 4th |  | 10% | 13% (-4; 32) | | 10% | 12% (-4; 32) | |
|  |  |  | highest |  | 2.9 mL | reference | | 2.9 mL | reference | |
|  | Continuous (per SD decrease) |  |  |  | 0% | 2% (-4; 7) | | 0% | 0% (0; 1) | |
|  | Concentration (mill/ml) | 327 | lowest |  | -17% | -4% (-30; 31) | | -17% | -3% (-29; 34) | |
|  |  |  | 2nd |  | 0% | 12% (-17; 51) | | 0% | 13% (-17; 53) | |
|  |  |  | 3rd |  | 1% | 22% (-4; 54) | | 1% | 22% (-4; 54) | |
|  |  |  | 4th |  | -18% | -8% (-29; 19) | | -18% | -8% (-30; 19) | |
|  |  |  | highest |  | 57 mill/mL | reference | | 57 mill/mL | reference | |
|  | Continuous (per SD decrease) |  |  |  | -3% | 2% (-6; 10) | | -3% | 2% (-6; 10) | |
|  | Total sperm count (mill)^f^ | 265 | lowest |  | -22% | -3% (-31; 37) | | -22% | -2% (-31; 39) | |
|  |  |  | 2nd |  | 1% | 18% (-17; 67) | | 1% | 18% (-17; 67) | |
|  |  |  | 3rd |  | 4% | 25% (-5; 66) | | 4% | 25% (-5; 65) | |
|  |  |  | 4th |  | 1% | 9% (-18; 45) | | 1% | 9% (-18; 45) | |
|  |  |  | highest |  | 163 mill | reference | | 163 mill | reference | |
|  | Continuous (per SD decrease) |  |  |  | -3% | 2% (-7; 11) | | -3% | 2% (-7; 12) | |
|  | Motility (modelled as NP+IM %)^g^ | 320 | lowest |  | -10% | -5% (-17; 8) | | -10% | -3% (-15; 11) | |
|  |  |  | 2nd |  | -9% | -7% (-19; 8) | | -9% | -5% (-18; 9) | |
|  |  |  | 3rd |  | -20% | -18% (-27; -7) | | -20% | -17% (-26; -6) | |
|  |  |  | 4th |  | -7% | -5% (-14; 7) | | -7% | -5% (-16; 7) | |
|  |  |  | highest |  | 45% | reference | | 45% | reference | |
|  | Continuous (per SD decrease) |  |  |  | -5% | -3% (-7; 0) | | -5% | -3% (-7; 1) | |
|  | Morphology (% normal) | 319 | lowest |  | -8% | -8% (-30; 22) | | -8% | -7% (-30; 23) | |
|  |  |  | 2nd |  | 13% | 17% (-7; 48) | | 13% | 18% (-8; 50) | |
|  |  |  | 3rd |  | 4% | 5% (-14; 29) | | 4% | 5% (-14; 29) | |
|  |  |  | 4th |  | -4% | -2% (-22; 22) | | -4% | -3% (-22; 21) | |
|  |  |  | highest |  | 6.7% | reference | | 6.7% | reference | |
|  | Continuous (per SD decrease) |  |  |  | 1% | 1% (-6; 8) | | 1% | 0% (-6; 8) | |
|  | DFI (%) | 304 | lowest |  | -2% | 2% (-16; 24) | | -2% | 5% (-15; 28) | |
|  |  |  | 2nd |  | 7% | 6% (-13; 29) | | 7% | 8% (-12; 31) | |
|  |  |  | 3rd |  | 2% | 7% (-10; 29) | | 2% | 7% (-10; 29) | |
|  |  |  | 4th |  | 4% | 16% (-5; 42) | | 4% | 15% (-5; 41) | |
|  |  |  | highest |  | 9.9% | reference | | 9.9% | reference | |
|  | Continuous (1-SD) |  |  |  | 0% | 1% (-5; 7) | | 0% | 2% (-4; 8) | |
|  | HDS (%) | 304 | lowest |  | 10% | 8% (-10; 29) | | 10% | 11% (-8; 34) | |
|  |  |  | 2nd |  | 5% | 2% (-17; 25) | | 5% | 4% (-15; 27) | |
|  |  |  | 3rd |  | -8% | -3% (-17; 13) | | -8% | -3% (-16; 13) | |
|  |  |  | 4th |  | 7% | 7% (-11; 28) | | 7% | 6% (-12; 27) | |
|  |  |  | highest |  | 9.8% | Reference | | 9.8% | Reference | |
|  | Continuous (per SD decrease) |  |  |  | 0% | 0% (-5; 5) | | 0% | 1% (-5; 6) | |
|  |  |  |  |  |  |  |  |  |  |  |
| Testicular volume^h^ | |  |  |  |  |  |  |  |  |  |
|  | Average testicular volume (ml) | 329 | lowest |  | 0% | 0% (-10; 11) | | 0% | 0% (-10; 12) | |
|  |  |  | 2nd |  | -10% | -12% (-21; -1) | | -10% | -11% (-21; -1) | |
|  |  |  | 3rd |  | 3% | 1% (-9; 11) | | 3% | 1% (-9; 11) | |
|  |  |  | 4th |  | -6% | -9% (-18; 1) | | -6% | -9% (-18; 0) | |
|  |  |  | highest |  | 17% | reference | | 17% | reference | |
|  | Continuous (per SD decrease) |  |  |  | 0% | -1% (-4; 2) | | 0% | -1% (-4; 2) | |
|  |  |  |  |  |  |  |  |  |  |  |
| Reproductive hormones^i^ | |  |  |  |  |  |  |  |  |  |
|  | Testosterone (nmol/L) | 330 | lowest |  | -4% | -4% (-13; 5) | | -4% | -4% (-13; 6) | |
|  |  |  | 2nd |  | -2% | -4% (-13; 5) | | -2% | -4% (-13; 5) | |
|  |  |  | 3rd |  | 4% | 3% (-7; 13) | | 4% | 3% (-6; 14) | |
|  |  |  | 4th |  | -4% | -6% (-14; 2) | | -4% | -6% (-14; 2) | |
|  |  |  | highest |  | 18.5 nmol/L | reference | | 18.5 nmol/L | reference | |
|  | Continuous (per SD decrease) |  |  |  | -1% | -2% (-4; 1) | | -1% | -1% (-3; 2) | |
|  | Estradiol (pmol/L) | 330 | lowest |  | -4% | -3% (-22; 20) | | -4% | 1% (-20; 26) | |
|  |  |  | 2nd |  | 0% | 6% (-12; 28) | | 0% | 9% (-10; 32) | |
|  |  |  | 3rd |  | 6% | 6% (-11; 25) | | 6% | 7% (-10; 27) | |
|  |  |  | 4th |  | -6% | -4% (-18; 12) | | -6% | -5% (-18; 11) | |
|  |  |  | highest |  | 51.7 pmol/L | reference | | 51.7 pmol/L | reference | |
|  | Continuous (per SD decrease) |  |  |  | 0% | 0% (-5; 6) | | 0% | 1% (-4; 7) | |
|  | SHBG (nmol/L) | 329 | lowest |  | -8% | -8% (-18; 4) | | -8% | -7% (-18; 5) | |
|  |  |  | 2nd |  | -6% | -6% (-16; 5) | | -6% | -6% (-16; 6) | |
|  |  |  | 3rd |  | 3% | 2% (-10; 15) | | 3% | 2% (-10; 15) | |
|  |  |  | 4th |  | -1% | -2% (-12; 9) | | -1% | -2% (-12; 9) | |
|  |  |  | highest |  | 35.5 nmol/L | reference | | 35.5 nmol/L | reference | |
|  | Continuous (per SD decrease) |  |  |  | -1% | -2% (-5; 2) | | -1% | -2% (-5; 2) | |
|  | FSH (IU/L) | 329 | lowest |  | -27% | -25% (-37; -10) | | -27% | -24% (-37; -8) | |
|  |  |  | 2nd |  | -5% | -2% (-18; 17) | | -5% | -1% (-18; 18) | |
|  |  |  | 3rd |  | -12% | -14% (-28; 3) | | -12% | -14% (-28; 3) | |
|  |  |  | 4th |  | 1% | 4% (-14; 26) | | 1% | 4% (-14; 26) | |
|  |  |  | highest |  | 4.3 IU/L | reference | | 4.3 IU/L | reference | |
|  | Continuous (per SD decrease) |  |  |  | -6% | -5% (-11; 0) | | -6% | -5% (-10; 1) | |
|  | LH (IU/L) | 329 | lowest |  | -6% | -7% (-18; 5) | | -6% | -7% (-18; 5) | |
|  |  |  | 2nd |  | 2% | 3% (-9; 18) | | 2% | 3% (-10; 18) | |
|  |  |  | 3rd |  | 0% | 1% (-10; 15) | | 0% | 1% (-10; 14) | |
|  |  |  | 4th |  | 6% | 4% (-8; 17) | | 6% | 4% (-7; 18) | |
|  |  |  | highest |  | 5.3 IU/L | reference | | 5.3 IU/L | reference | |
|  | Continuous (per SD decrease) |  |  |  | 0% | 0% (-3; 4) | | 0% | 0% (-3; 4) | |
|  | Free testosterone (nmol/L) | 329 | lowest |  | -1% | -2% (-10; 8) | | -1% | -1% (-10; 8) | |
|  |  |  | 2nd |  | 1% | -3% (-11; 8) | | 1% | -2% (-11; 8) | |
|  |  |  | 3rd |  | 2% | 1% (-7; 10) | | 2% | 1% (-7; 10) | |
|  |  |  | 4th |  | -4% | -7% (-14; 1) | | -4% | -7% (-13; 1) | |
|  |  |  | highest |  | 0.4 nmol/L | reference | | 0.4 nmol/L | reference | |
|  | Continuous (per SD decrease) |  |  |  | 0% | -1% (-4; 2) | | 0% | -1% (-4; 2) | |
| Abbreviations: DFE, dietary folate equivalents; SD, standard deviation; DFI, DNA fragmentation index; HDS, High DNA stainability; SHBG, Sex-hormone binding globulin; FSH, Follicle-stimulating hormone; LH, Luteinizing hormone; BMI, body mass index; TTP, time to pregnancy; MAR, medically assisted reproduction; HEI, healthy eating index | | | | | | | | | | |
|  |  |  |  |  |  |  |  |  |  |  |
| ^a^ Adjusted for maternal age at delivery, highest parental social class, maternal first trimester smoking, maternal pre-pregnancy BMI, TTP including MAR. Only estimates in B adjusted for maternal healthy eating index | | | | | | | | | | |
|  |  |  |  |  |  |  |  |  |  |  |
| ^b^ Total folate intake expressed as dietary folate equivalents (DFEs) calculated as DFE = folate from food in μg/day + folic acid from vitamins in μg/day × 1.7 | | | | | | | | |  |  |
| ^c^ SD = 341 µg/day | |  |  |  |  |  |  |  |  |  |
| ^d^ The numbers are from the adjusted model and vary due to the exclusion of azoosperme semen samples and due to potential missingness on covariates | | | | | | | | |  |  |
| ^e^ Further adjusted for abstinence time, spillage and place of semen sample collection | | | | | |  |  |  |  |  |
| ^f^ Excluding samples with spillage | |  |  |  |  |  |  |  |  |  |
| ^g^ Further adjusted for interval from ejaculation to analysis of motility. Estimates represents the relative difference in the proportion of non-progressive and immotile spermatozoa. Therefore, positive estimates should be interpreted as a relatively lower progressive motility and vice versa | | | | | | | | | | |
|  |  |  |  |  |  |  |  |  |  |  |
| ^h^ Adjusted for abstinence time | |  |  |  |  |  |  |  |  |  |
| ^i^ Adjusted for time of blood sample drawing | |  |  |  |  |  |  |  |  |  |

| **Supplementary Table 2. Sub analysis.** Crude and adjusted^a^ (95% confidence intervals) relative percentage differences in markers of male fecundity according to prenatal exposure to folic acid from supplements in μg/day in categories in 787 participants from the fetal programming of semen quality (FEPOS) cohort, Denmark, 1998 - 2019. | | | | | | | |
| --- | --- | --- | --- | --- | --- | --- | --- |
|  |  |  |  |  |  |  |  |
|  |  |  |  |  |  |  |  |
|  |  |  |  |  |  |  |  |
|  |  |  |  |  |  |  |  |
|  |  |  |  |  |  |  |  |
|  |  |  |  |  | Folic acid from supplements^b^ | | |
|  |  |  |  |  |  |  |  |
|  |  | n^c^ |  |  | Crude | Adjusted (95% CI) | |
|  |  |  |  |  |  |  |  |
| Semen characteristics^d^ | |  |  |  |  |  |  |
|  | Volume (ml)^e^ | 615 | 0 µg/day |  | 0% | -3% (-11; 6) | |
|  |  |  | 0 – 399 µg/day |  | 15% | 14% (1; 29) | |
|  |  |  | ≥ 400 µg/day |  | 2.9 mL | reference | |
|  | Concentration (mill/ml) | 752 | 0 µg/day |  | 0% | 7% (-8; 24) | |
|  |  |  | 0 – 399 µg/day |  | 7% | 6% (-16; 34) | |
|  |  |  | ≥ 400 µg/day |  | 50 mill/mL | reference | |
|  | Total sperm count (mill)^e^ | 615 | 0 µg/day |  | -11% | -8% (-23; 9) | |
|  |  |  | 0 – 399 µg/day |  | 3% | 6% (-17; 35) | |
|  |  |  | ≥ 400 µg/day |  | 156 mill | reference | |
|  | Motility (modelled as NP+IM %)^f^ | 734 | 0 µg/day |  | -17% | -15% (-21; -9) | |
|  |  |  | 0 – 399 µg/day |  | -7% | -9% (-18; 0) | |
|  |  |  | ≥ 400 µg/day |  | 44% | reference | |
|  | Morphology (% normal) | 732 | 0 µg/day |  | 6% | 9% (-6; 26) | |
|  |  |  | 0 – 399 µg/day |  | 6% | 5% (-13; 26) | |
|  |  |  | ≥ 400 µg/day |  | 6.5% | reference | |
|  | DFI (%) | 703 | 0 µg/day |  | 17% | 17% (3; 32) | |
|  |  |  | 0 – 399 µg/day |  | -4% | -6% (-20; 11) | |
|  |  |  | ≥ 400 µg/day |  | 9.4% | reference | |
|  | HDS (%) | 703 | 0 µg/day |  | 6% | 5% (-6; 18) | |
|  |  |  | 0 – 399 µg/day |  | -1% | 1% (-13; 18) | |
|  |  |  | ≥ 400 µg/day |  | 9.9% | reference | |
|  |  |  |  |  |  |  |  |
| Testicular volume^g^ | |  |  |  |  |  |  |
|  | Average testicular volume (ml) | 758 | 0 µg/day |  | -8% | -9% (-15; -4) | |
|  |  |  | 0 – 399 µg/day |  | -6% | -6% (-15; 3) | |
|  |  |  | ≥ 400 µg/day |  | 17% | reference | |
|  |  |  |  |  |  |  |  |
| Reproductive hormones^h^ | |  |  |  |  |  |  |
|  | Testosterone (nmol/L) | 758 | 0 µg/day |  | 0% | -1% (-7; 5) | |
|  |  |  | 0 – 399 µg/day |  | -2% | -4% (-11; 5) | |
|  |  |  | ≥ 400 µg/day |  | 18.7 nmol/L | reference | |
|  | Estradiol (pmol/L) | 758 | 0 µg/day |  | 11% | 12% (1; 25) | |
|  |  |  | 0 – 399 µg/day |  | 1% | 3% (-12; 19) | |
|  |  |  | ≥ 400 µg/day |  | 51.0 pmol/L | reference | |
|  | SHBG (nmol/L) | 757 | 0 µg/day |  | -2% | -3% (-10; 4) | |
|  |  |  | 0 – 399 µg/day |  | -4% | -5% (-15; 5) | |
|  |  |  | ≥ 400 µg/day |  | 35.0 nmol/L | reference | |
|  | FSH (IU/L) | 757 | 0 µg/day |  | 0% | 0% (-13; 14) | |
|  |  |  | 0 – 399 µg/day |  | -10% | -12% (-25; 3) | |
|  |  |  | ≥ 400 µg/day |  | 4.2 IU/L | reference | |
|  | LH (IU/L) | 757 | 0 µg/day |  | 6% | 6% (-2; 15) | |
|  |  |  | 0 – 399 µg/day |  | 0% | -1% (-11; 10) | |
|  |  |  | ≥ 400 µg/day |  | 5.3 IU/L | reference | |
|  | Free testosterone (nmol/L) | 757 | 0 µg/day |  | 1% | 0% (-6; 6) | |
|  |  |  | 0 – 399 µg/day |  | 0% | -1% (-9; 7) | |
|  |  |  | ≥ 400 µg/day |  | 0.4 nmol/L | reference | |
| Abbreviations: SD, standard deviation; DFI, DNA fragmentation index; HDS, High DNA stainability; SHBG, Sex-hormone binding globulin; FSH, Follicle-stimulating hormone; LH, Luteinising hormone; BMI, body mass index; TTP, time to pregnancy; MAR, medically assisted reproduction | | | | | | | |
|  |  |  |  |  |  |  |  |
|  |  |  |  |  |  |  |  |
| ^a^ Adjusted for maternal age at delivery, highest parental social class, maternal 1. trimester smoking, maternal pre-pregnancy BMI, TTP including MAR and folate from diet | | | | | | | |
|  |  |  |  |  |  |  |  |
| ^b^ Assessed in mid-pregnancy | |  |  |  |  |  |  |
| ^c^ The numbers are from the adjusted model and vary due to the exclusion of azoosperme semen samples and due to potential missingness on covariates | | | | | | | |
|  |  |  |  |  |  |  |  |
| ^d^ Further adjusted for abstinence time, spillage and place of semen sample collection | | | |  |  |  |  |
| ^e^ Excluding samples with spillage | |  |  |  |  |  |  |
| ^f^ Further adjusted for interval from ejaculation to analysis of motility. Estimates represents the relative difference in the proportion of non-progressive and immotile spermatozoa. Therefore, positive estimates should be interpreted as a relatively lower progressive motility and vice versa | | | | | | | |
|  |  |  |  |  |  |  |  |
| ^g^ Further adjusted for abstinence time | |  |  |  |  |  |  |
| ^h^ Further adjusted for time of blood sample drawing | |  |  |  |  |  |  |

| **Supplementary Table 3. Sub analysis.** Crude and adjusted^a^ (95% confidence intervals) relative percentage differences in markers of male fecundity according to prenatal exposure to folate from diet in μg/day in quintiles and per standard deviation decrease^b^ in 787 participants from the fetal programming of semen quality (FEPOS) cohort, Denmark, 1998 - 2019. | | | | | | | |
| --- | --- | --- | --- | --- | --- | --- | --- |
|  |  |  |  |  |  |  |  |
|  |  |  |  |  |  |  |  |
|  |  |  |  |  |  |  |  |
|  |  |  |  |  |  |  |  |
|  |  |  |  |  |  |  |  |
|  |  |  |  |  | Folate from diet^c^ | | |
|  |  |  |  |  |  |  |  |
|  |  |  |  |  | Crude | Adjusted (95% CI) | |
|  |  |  |  |  |  |  |  |
| Semen characteristics^e^ | | n^d^ |  |  |  |  |  |
|  | Volume (ml)^f^ | 615 | lowest |  | -12% | -10% (-20; 1) | |
|  |  |  | 2nd |  | 0% | 2% (-9; 15) | |
|  |  |  | 3rd |  | -3% | -4% (-14; 6) | |
|  |  |  | 4th |  | -5% | -5% (-15; 7) | |
|  |  |  | highest |  | 3.0 mL | reference | |
|  | Continuous (per SD decrease) |  |  |  | -3% | -2% (-5; 1) | |
|  | Concentration (mill/ml) | 752 | lowest |  | -15% | -15% (-29; 3) | |
|  |  |  | 2nd |  | -11% | -12% (-28; 6) | |
|  |  |  | 3rd |  | -5% | -7% (-23; 13) | |
|  |  |  | 4th |  | 1% | -7% (-22; 12) | |
|  |  |  | highest |  | 53 mill/mL | reference | |
|  | Continuous (per SD decrease) |  |  |  | -5% | -3% (-19; 14) | |
|  | Total sperm count (mill)^f^ | 615 | lowest |  | -16% | -17% (-32; 2) | |
|  |  |  | 2nd |  | -9% | -7% (-25; 17) | |
|  |  |  | 3rd |  | 4% | 2% (-17; 24) | |
|  |  |  | 4th |  | -1% | -7% (-25; 15) | |
|  |  |  | highest |  | 151 mill | reference | |
|  | Continuous (per SD decrease) |  |  |  | -6% | -4% (-11; 4) | |
|  | Motility (modelled as NP+IM %)^g^ | 734 | lowest |  | 400% | 4% (-5; 14) | |
|  |  |  | 2nd |  | -4% | -6% (-14; 4) | |
|  |  |  | 3rd |  | 0% | -1% (-10; 10) | |
|  |  |  | 4th |  | 1% | -1% (-10; 10) | |
|  |  |  | highest |  | 38% | reference | |
|  | Continuous (per SD decrease) |  |  |  | 0% | 0% (-3; 2) | |
|  | Morphology (% normal) | 732 | lowest |  | 2% | 4% (-11; 21) | |
|  |  |  | 2nd |  | -5% | -4% (-18; 14) | |
|  |  |  | 3rd |  | 3% | 7% (-9; 26) | |
|  |  |  | 4th |  | 2% | 4% (-11; 21) | |
|  |  |  | highest |  | 6.8% | reference | |
|  | Continuous (per SD decrease) |  |  |  | 0% | 1% (-4; 6) | |
|  | DFI (%) | 703 | lowest |  | -15% | -21% (-31; -9) | |
|  |  |  | 2nd |  | -11% | -18% (-27; -6) | |
|  |  |  | 3rd |  | -6% | -7% (-19; 6) | |
|  |  |  | 4th |  | -4% | -10% (-21; 2) | |
|  |  |  | highest |  | 11.4% | reference | |
|  | Continuous (1-SD) |  |  |  | -4% | -4% (-9; 1) | |
|  | HDS (%) | 703 | lowest |  | 1% | -1% (-13; 12) | |
|  |  |  | 2nd |  | 2% | -1% (-13; 13) | |
|  |  |  | 3rd |  | 2% | -1% (-13; 13) | |
|  |  |  | 4th |  | -2% | -2% (-14; 11) | |
|  |  |  | highest |  | 10 | Reference | |
|  | Continuous (per SD decrease) |  |  |  | -1% | -1% (-5; 2) | |
|  |  |  |  |  |  |  |  |
| Testicular volume^h^ | |  |  |  |  |  |  |
|  | Average testicular volume (ml) | 758 | lowest |  | -4% | -3% (-11; 5) | |
|  |  |  | 2nd |  | 0% | -4% (-11; 4) | |
|  |  |  | 3rd |  | -6% | -8% (-15; 0) | |
|  |  |  | 4th |  | 0% | 0% (-8; 8) | |
|  |  |  | highest |  | 16% | reference | |
|  | Continuous (per SD decrease) |  |  |  | -2% | -2% (-4; 0) | |
|  |  |  |  |  |  |  |  |
| Reproductive hormones^i^ | |  |  |  |  |  |  |
|  | Testosterone (nmol/L) | 758 | lowest |  | -2% | -1% (-8; 5) | |
|  |  |  | 2nd |  | 1% | 0% (-6; 7) | |
|  |  |  | 3rd |  | -5% | -5% (-11; 1) | |
|  |  |  | 4th |  | 2% | % (-6; 7) | |
|  |  |  | highest |  | 18.8 nmol/L | reference | |
|  | Continuous (per SD decrease) |  |  |  | -1% | -1% (-2; 1) | |
|  | Estradiol (pmol/L) | 758 | lowest |  | 1% | -2% (-13; 12) | |
|  |  |  | 2nd |  | 2% | 2% (-10; 15) | |
|  |  |  | 3rd |  | -4% | -5% (-15; 7) | |
|  |  |  | 4th |  | 1% | 0% (-12; 12) | |
|  |  |  | highest |  | 55.0 pmol/L | reference | |
|  | Continuous (per SD decrease) |  |  |  | 0% | -1% (-4; 3) | |
|  | SHBG (nmol/L) | 757 | lowest |  | -5% | -3% (-11; 5) | |
|  |  |  | 2nd |  | -1% | -2% (-10; 6) | |
|  |  |  | 3rd |  | -3% | -4% (-12; 5) | |
|  |  |  | 4th |  | 2% | 1% (-7; 9) | |
|  |  |  | highest |  | 34.9 nmol/L | reference | |
|  | Continuous (per SD decrease) |  |  |  | -3% | -2% (-4; 0) | |
|  | FSH (IU/L) | 757 | lowest |  | -11% | -9% (-25; 11) | |
|  |  |  | 2nd |  | 3% | 0% (-19; 23) | |
|  |  |  | 3rd |  | 4% | 3% (-15; 25) | |
|  |  |  | 4th |  | 7% | 1% (-18; 24) | |
|  |  |  | highest |  | 4.1 IU/L | reference | |
|  | Continuous (per SD decrease) |  |  |  | 0% | 1% (-3; 6) | |
|  | LH (IU/L) | 757 | lowest |  | -5% | -5% (-15; 6) | |
|  |  |  | 2nd |  | 4% | 6% (-6; 19) | |
|  |  |  | 3rd |  | -3% | -2% (-12; 9) | |
|  |  |  | 4th |  | 1% | 1% (-11; 13) | |
|  |  |  | highest |  | 5.5 IU/L | reference | |
|  | Continuous (per SD decrease) |  |  |  | 0% | 0% (-2; 3) | |
|  | Free testosterone (nmol/L) | 757 | lowest |  | 0% | -1% (-7; 6) | |
|  |  |  | 2nd |  | 1% | 1% (-5; 8) | |
|  |  |  | 3rd |  | -3% | -3% (-9; 4) | |
|  |  |  | 4th |  | 0% | 0% (-6; 7) | |
|  |  |  | highest |  | 0.4 nmol/L | reference | |
|  | Continuous (per SD decrease) |  |  |  | 0% | 0% (-1; 2) | |
| Abbreviations: SD, standard deviation; DFI, DNA fragmentation index; HDS, High DNA stainability; SHBG, Sex-hormone binding globulin; FSH, Follicle-stimulating hormone; LH, Luteinizing hormone; BMI, body mass index; TTP, time to pregnancy; MAR, medically assisted reproduction; FFQ, food frequency questionnaire | | | | | | | |
|  |  |  |  |  |  |  |  |
|  |  |  |  |  |  |  |  |
| ^a^ Adjusted for maternal age at delivery, highest parental social class, maternal 1. trimester smoking, maternal pre-pregnancy BMI, TTP including MAR and folic acid from supplements | | | | | | | |
|  |  |  |  |  |  |  |  |
| ^b^ SD = 122 µg/day | |  |  |  |  |  |  |
| ^c^ Assessed in mid-pregnancy from the FFQ | |  |  |  |  |  |  |
| ^d^ The numbers are from the adjusted model and vary due to the exclusion of azoosperme semen samples and due to potential missingness on covariates | | | | | | | |
|  |  |  |  |  |  |  |  |
| ^e^ Further adjusted for abstinence time, spillage and place of semen sample collection | | | | | |  |  |
| ^f^ Excluding samples with spillage | |  |  |  |  |  |  |
| ^g^ Further adjusted for interval from ejaculation to analysis of motility. Estimates represents the relative difference in the proportion of non-progressive and immotile spermatozoa. Therefore, positive estimates should be interpreted as a relatively lower progressive motility and vice versa | | | | | | | |
|  |  |  |  |  |  |  |  |
| ^h^ Further adjusted for abstinence time | |  |  |  |  |  |  |
| ^i^ Further adjusted for time of blood sample drawing | |  |  |  |  |  |  |

| **Supplementary Table 4.** Baseline characteristics according to supplement intake reported in early first trimester in 1,058 participants from the fetal programming of semen quality (FEPOS) cohort, Denmark, 1998 - 2019 | | | | | | | | | | | |  |
| --- | --- | --- | --- | --- | --- | --- | --- | --- | --- | --- | --- | --- |
|  |  |  |  |  |  |  |  |  |  |  |  |  |
|  |  | Supplement intake | | | | | | | |  |  | |
|  |  | No | | Other | | Multi | | Folate | | Missings | |  |
|  |  | No. | % | No. | % | No. | % | No. | % | No. | % | |
|  |  | 205 | 19 | 56 | 5 | 552 | 52 | 245 | 23 |  |  | |
| **Mid-pregnancy intake of folate and folic acid** | |  |  |  |  |  |  |  |  |  |  | |
| Total DFEs^a^: p50 (range) | | 390 (149-1,206) | | 373 (181-978) | | 401 (105-1,014) | | 414 (129-1,467) | | 271 (26%) | |  |
| Folate from diet: p50 (range) | | 336 (24-640) | | 343 (181-514) | | 350 (105-1,107) | | 362 (129-613) | | 271 (26%) | |  |
| Folic acid from supplements: p50 (range) | | 0 (0-400) | | 0 (0-377) | | 0 (0-720) | | 0 (0-600) | | 271 (26%) | |  |
|  |  |  |  |  |  |  |  |  |  |  |  | |
| Maternal baseline characteristics | |  |  |  |  |  |  |  |  |  |  | |
| Highest social class of parents | |  |  |  |  |  |  |  |  | 0 | 0 | |
|  | High-grade professional | 48 | 23 | 15 | 27 | 196 | 36 | 100 | 41 |  |  | |
|  | Low-grade professional | 69 | 34 | 21 | 38 | 177 | 32 | 83 | 34 |  |  | |
|  | Skilled or unskilled worker | 76 | 37 | > 15^a^ | > 27^a^ | 155 | 28 | 51 | 21 |  |  | |
|  | Student / economically inactive | 12 | 6 | < 5^a^ | < 9^a^ | 24 | 4 | 11 | 4 |  |  | |
| Maternal age at delivery (years) | | 30.5 (4.0) | | 31.5 (3.7) | | 31.0 (4.3) | | 31.0 (4.2) | | < 5^a^ | 0 | |
| Maternal pre-pregnancy BMI (kg/m^2^) | |  |  |  |  |  |  |  |  | 25 | 2 | |
|  | < 18.5 | 8 | 4 | < 5^a^ | < 9^a^ | 31 | 6 | 22 | 9 |  |  | |
|  | 18.5-24.9 | 134 | 65 | 42 | 75 | 403 | 73 | 179 | 73 |  |  | |
|  | 25-29.9 | 36 | 18 | 7 | 13 | 88 | 16 | 33 | 13 |  |  | |
|  | > 30 | 16 | 8 | < 5^a^ | < 9^a^ | 22 | 4 | 5 | 2 |  |  | |
| Maternal smoking 1. trimester (cigarettes/day) | | |  |  |  |  |  |  |  | 0 | 0 | |
|  | 0 | 145 | 71 | 42 | 75 | 429 | 78 | 199 | 81 |  |  | |
|  | 1-10 | 49 | 24 | > 9^a^ | > 16^a^ | 105 | 19 | 39 | 16 |  |  | |
|  | > 10 | 11 | 5 | < 5^a^ | < 9^a^ | 18 | 3 | 7 | 3 |  |  | |
| TTP incl. unplanned pregnancy and MAR | |  |  |  |  |  |  |  |  | 6 | 1 | |
|  | Unplanned pregnancy | 40 | 20 | 8 | 14 | 96 | 17 | 31 | 13 |  |  | |
|  | TTP 0-5 months | < 113^a^ | < 56^a^ | 38 | 68 | < 337^a^ | < 62^a^ | < 149^a^ | < 61^a^ |  |  | |
|  | TTP 6-12 months | 23 | 11 | < 5^a^ | < 9^a^ | 58 | 11 | 22 | 9 |  |  | |
|  | TTP > 12 months or MAR | 29 | 14 | 5 | 9 | 61 | 11 | 43 | 18 |  |  | |
|  |  |  |  |  |  |  |  |  |  |  |  | |
| Precision variables | |  |  |  |  |  |  |  |  |  |  | |
| Abstinence time (days) | | 2.3 (1.5) | | 1.9 (0.9) | | 2.4 (1.6) | | 2.3 (1.3) | | 5 | 0 | |
| Place of semen sample collection | |  |  |  |  |  |  |  |  | 10 | 1 | |
|  | At home | 28 | 14 | 6 | 11 | 69 | 13 | 35 | 14 |  |  | |
|  | In the clinic | > 177^a^ | > 86^a^ | 50 | 89 | 477 | 86 | > 210^a^ | > 86^a^ |  |  | |
| Spillage | |  |  |  |  |  |  |  |  | 9 | 1 | |
|  | Yes | 38 | 19 | 8 | 14 | 98 | 18 | 38 | 16 |  |  | |
|  | No | > 167^a^ | > 81^a^ | 48 | 86 | > 454^a^ | > 82^a^ | > 207^a^ | > 84^a^ |  |  | |
| Interval ejaculation - analysis (min) | | 51.0 (21.0) | | 45.5 (18.0) | | 50.0 (19.0) | | 51.0 (19.0) | | 12 | 1 | |
| Time at blood sample collection | |  |  |  |  |  |  |  |  | 11 | 1 | |
|  | morning < 12 p.m. | 82 | 40 | 24 | 43 | 185 | 34 | 86 | 35 |  |  | |
|  | afternoon 12-18 p.m. | 100 | 49 | 28 | 50 | 297 | 54 | > 136^a^ | > 56^a^ |  |  | |
|  | evening > 18 p.m. | 23 | 11 | < 5^a^ | < 9^a^ | 61 | 11 | 23 | 9 |  |  | |
| Numbers in table reported as n (%), or mean (SD). Percentage may not add up due to rounding to the nearest number | | | | | | | |  |  |  |  | |
| Abbreviations: BMI, body mass index; SD, standard deviation; TTP, time to pregnancy; MAR, medically assisted reproduction | | | | | | | |  |  |  |  | |
| ^a^ Due to local data regulations it is not allowed to report numbers smaller than five, why the numbers in the table have been changed to mask the numbers smaller than five | | | | | | | | | | | |  |

| **Supplementary Table 5.** Markers of male fecundity^a^ according to supplement intake reported in early first trimester in 1,058 participants from the fetal programming of semen quality (FEPOS) cohort, Denmark, 1998 - 2019 | | | | | | | | | | | |
| --- | --- | --- | --- | --- | --- | --- | --- | --- | --- | --- | --- |
|  |  |  |  |  |  |  |  |  |  |  |  |
|  |  | Vitamin intake | | | | | | | |  |  |
|  |  | No | | Other | | Multi | | Folate | | Missings | |
|  |  | No. | % | No. | % | No. | % | No. | % | No. | % |
|  |  | 205 | 19 | 56 | 5 | 552 | 52 | 245 | 23 |  |  |
|  |  |  |  |  |  |  |  |  |  |  |  |
| Semen quality characteristics | |  |  |  |  |  |  |  |  |  |  |
|  | Volume (ml)^c^ | 2.6 (1.8-3.4) | | 2.6 (1.9-3.3) | | 2.8 (1.9-3.8) | | 2.7 (2.0-3.6) | | 192 | 18 |
|  | Concentration (mill/ml) | 44 (21-77) | | 42 (21-72) | | 35 (17-67) | | 41 (21-76) | | 6 | 0.6 |
|  | Total sperm count (mill)^c^ | 105 (44-194) | | 119 (47-214) | | 98 (44-192) | | 113 (53-213) | | 192 | 18 |
|  | Motility (PR %)^d^ | 64 (55-74) | | 62 (52-74) | | 63 (52-73) | | 65 (52-74) | | 23 | 2.2 |
|  | Morphology (% normal)^d^ | 6 (4-10) | | 6 (3-10) | | 6 (2-10) | | 7 (4-11) | | 29 | 2.7 |
|  | DFI (%) | 9 (7-13) | | 9 (7-14) | | 10 (6-14) | | 10 (7-14) | | 73 | 6.9 |
|  | HDS (%) | 10 (7-14) | | 9 (7-13) | | 9 (7-13) | | 9 (7-13) | | 73 | 6.9 |
| Testicular volume (ml) | | 15 (11-20) | | 18 (13-22) | | 15 (12-20) | | 15 (12-20) | | < 5^b^ | 0.5 |
| Reproductive hormones | |  |  |  |  |  |  |  |  |  |  |
|  | Testosterone (nmol/L) | 18 (15-22) | | 18 (15-23) | | 18 (15-22) | | 18 (15-22) | | 12 | 1.1 |
|  | Estradiol (pmol/L) | 52 (34-72) | | 52 (36-70) | | 52 (34-73) | | 54 (36-76) | | 12 | 1.1 |
|  | SHBG (nmol/L) | 32 (26-42) | | 34 (27-41) | | 33 (26-41) | | 32 (24-42) | | 13 | 1.2 |
|  | FSH (IU/L) | 3.4 (2.4-5.1) | | 3.0 (2.1-5.4) | | 3.6 (2.6-5.1) | | 3.5 (2.5-4.9) | | 13 | 1.2 |
|  | LH (IU/L) | 4.9 (4.1-6.3) | | 4.8 (3.8-6.6) | | 5.1 (3.8-6.6) | | 5.2 (4.2-6.7) | | 13 | 1.2 |
|  | Free testosterone (nmol/L) | 0.38 (0.32-0.46) | | 0.39 (0.29-0.46) | | 0.38 (0.32-0.46) | | 0.39 (0.32-0.46) | | 13 | 1.2 |
| Abbreviations: p50, 50^th^ pseudo percentile; IQR, pseudo intra quartile range; PR, progressive motility; DFI, DNA fragmentation index; HDS, high DNA stainability; FSH, follicle-stimulating hormone; LH, luteinizing hormone | | | | | | | | | | | |
|  |  |  |  |  |  |  |  |  |  |  |  |
| ^a^ Reported as 50^th^ percentile (IQR). All percentiles are pseudo percentiles calculated from the average of five values | | | | | | |  |  |  |  |  |
| ^b^ Due to local data regulations it is not allowed to report numbers smaller than five, why the numbers in the table have been changed to mask the numbers smaller than five. | | | | | | | | | | |  |
| ^c^ Excluding samples from participants reporting spillage | | |  |  |  |  |  |  |  |  |  |
| ^d^ Excluding azoosperme samples | |  |  |  |  |  |  |  |  |  |  |

| **Supplementary Table 6. Sub analysis.** Crude and adjusted^a^ (95% confidence intervals) relative percentage differences in markers of male fecundity according to early pregnancy supplement intake. No supplement intake, intake of a supplement containing other vitamins and intake of multivitamins relative to intake of folic acid in 1,058 participants from the fetal programming of semen quality (FEPOS) cohort, Denmark. | | | | | | | | | | | | | | |
| --- | --- | --- | --- | --- | --- | --- | --- | --- | --- | --- | --- | --- | --- | --- |
|  |  |  |  |  |  |  |  |  |  |  |  |  |  |  |
|  |  |  |  |  |  |  |  |  |  |  |  |  |  |  |
|  |  |  |  |  |  |  |  |  |  |  |  |  |  |  |
|  |  |  |  |  |  |  |  |  |  |  |  |  |  |  |
|  |  |  |  | No supplement | | |  | Other supplement | | |  | Multivitamins | | |
|  |  | n^b^ |  | Crude | Adjusted (95% CI) | |  | Crude | Adjusted (95% CI) | |  | Crude | Adjusted (95% CI) | |
|  |  |  |  |  |  |  |  |  |  |  |  |  |  |  |
| Semen quality characteristics^c^ | |  |  |  |  |  |  |  |  |  |  |  |  |  |
|  | Volume (ml)^d^ | 835 |  | 0% | 1% (-9; 12) | |  | -5% | -7% (-19; 8) | |  | 5% | 5% (-2; 14) | |
|  | Concentration (mill/ml) | 1008 |  | 0% | 8% (-8; 26) | |  | -8% | 1% (-18; 25) | |  | -9% | -9% (-19; 4) | |
|  | Total sperm count (mill)^d^ | 835 |  | -7% | -3% (-18; 15) | |  | -4% | 10% (-16; 42) | |  | -8% | -8% (-20; 6) | |
|  | Motility (modelled as NP+IM %)^e^ | 985 |  | -3% | -5% (-13; 3) | |  | 0% | 0% (-12; 14) | |  | 3% | 1% (-5; 8) | |
|  | Morphology (% normal) | 985 |  | -4% | -3% (-15; 11) | |  | -11% | -8% (-25; 13) | |  | -10% | -11% (-20; -1) | |
|  | DFI (%) | 938 |  | -7% | -5% (-15; 7) | |  | -8% | -5% (-18; 11) | |  | -5% | .3% (-11; 5) | |
|  | HDS (%) | 938 |  | 9% | 6% (-6; 18) | |  | 1% | 0% (-14; 16) | |  | 2% | 3% (-5; 12) | |
| Testicular volume (ml)^f^ | | 1018 |  | 0% | -2% (-8; 6) | |  | 8% | 5% (-5; 16) | |  | 0% | -1% (-6; 4) | |
| Reproductive hormones^g^ | |  |  |  |  |  |  |  |  |  |  |  |  |  |
|  | Testosterone (nmol/L) | 1014 |  | 1% | 1% (-5; 7) | |  | -1% | -1% (-10; 8) | |  | -1% | -1% (-5; 4) | |
|  | Estradiol (pmol/L) | 1014 |  | -3% | -5% (-15; 5) | |  | -4% | -7% (-20; 9) | |  | -2% | -3% (-11; 6) | |
|  | SHBG (nmol/L) | 1013 |  | -1% | 0% (-8; 9) | |  | 1% | 0% (-10; 11) | |  | -1% | -1% (-7; 6) | |
|  | FSH (IU/L) | 1013 |  | 2% | 3% (-8; 16) | |  | 0% | -1% (-20; 22) | |  | 7% | 9% (-1; 21) | |
|  | LH (IU/L) | 1013 |  | -4% | -4% (-11; 2) | |  | -8% | -9% (-18; 1) | |  | -1% | 1% (-5; 7) | |
|  | Free testosterone (nmol/L) | 1013 |  | 2% | 2% (-4; 7) | |  | -1% | -2% (-10; 8) | |  | -1% | -1% (-5; 3) | |
|  |  |  |  |  |  |  |  |  |  |  |  |  |  |  |
| Abbreviations: NP, non-progressive motility; IM, Immotile; DFI, DNA fragmentation index; HDS, High DNA stainability; SHBG, Sex-hormone binding globulin; FSH, Follicle-stimulating hormone; LH, Luteinizing hormone; BMI, body mass index; TTP, time to pregnancy; MAR, medically assisted reproduction | | | | | | | | | | | | | | |
|  |  |  |  |  |  |  |  |  |  |  |  |  |  |  |
| ^a^ Adjusted for maternal age at delivery, highest parental social class, maternal 1. trimester smoking, maternal pre-pregnancy BMI, TTP including MAR | | | | | | | | | | | | | |  |
| ^b^ The numbers are from the adjusted model and vary due to the exclusion of azoosperme semen samples and due to potential missingness on covariates. | | | | | | | | | | | | | |  |
| ^c^ Further adjusted for abstinence time, spillage and place of semen sample collection. | | | | | | |  |  |  |  |  |  |  |  |
| ^d^ Excluding samples with spillage | |  |  |  |  |  |  |  |  |  |  |  |  |  |
| ^e^ Further adjusted for interval from ejaculation to analysis of motility. Estimates represents the relative difference in the proportion of non-progressive and immotile spermatozoa. Therefore, positive estimates should be interpreted as a relatively lower progressive motility and vice versa | | | | | | | | | | | | | | |
|  |  |  |  |  |  |  |  |  |  |  |  |  |  |  |
| ^f^ Further adjusted for abstinence time | |  |  |  |  |  |  |  |  |  |  |  |  |  |
| ^g^ Further adjusted for time of blood sample drawing | | |  |  |  |  |  |  |  |  |  |  |  |  |

**Supplementary figure 1.**


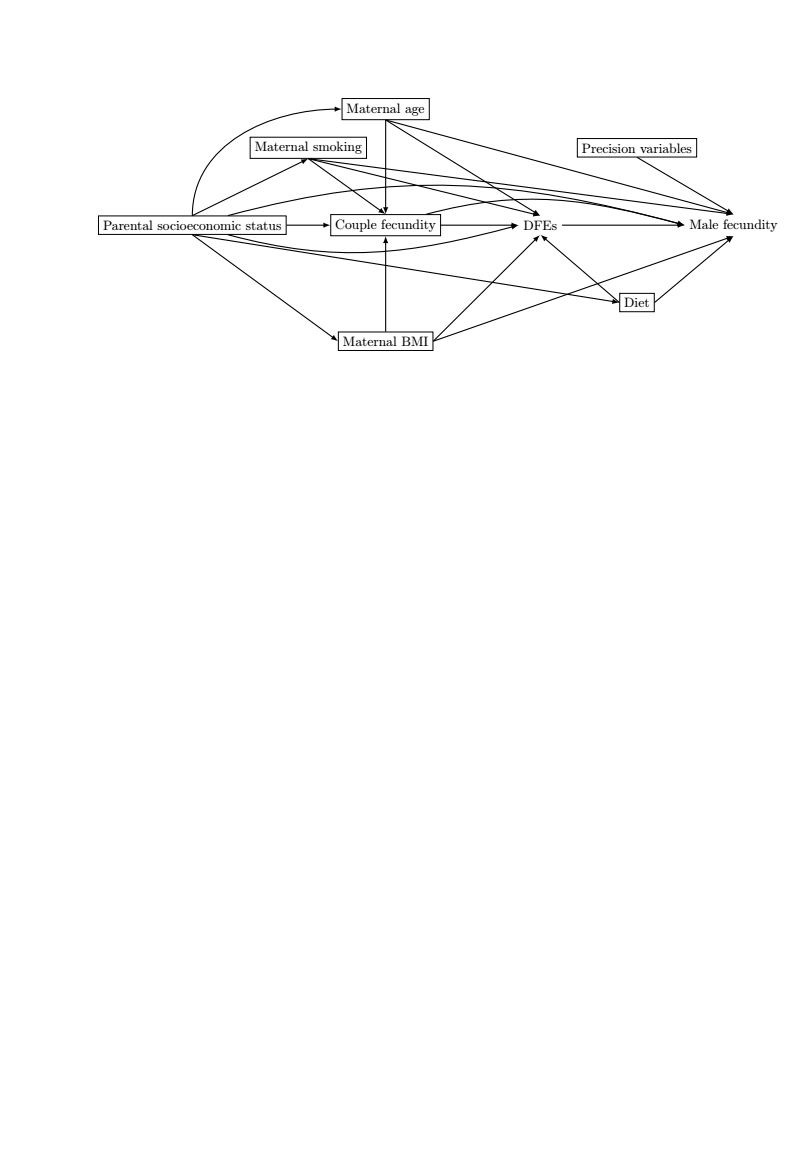


**Supplementary figure 1. Directed acyclic graph illustrating the proposed causal framework underlying this study.** Boxes indicate conditioning.

Abbreviations: BMI, body mass index. DFEs, dietary folate equivalents.
